# A novel multi-network approach reveals tissue-specific cellular modulators of fibrosis in systemic sclerosis

**DOI:** 10.1186/s13073-017-0417-1

**Published:** 2017-03-23

**Authors:** Jaclyn N. Taroni, Casey S. Greene, Viktor Martyanov, Tammara A. Wood, Romy B. Christmann, Harrison W. Farber, Robert A. Lafyatis, Christopher P. Denton, Monique E. Hinchcliff, Patricia A. Pioli, J. Matthew Mahoney, Michael L. Whitfield

**Affiliations:** 1 Department of Molecular and Systems Biology, Geisel School of Medicine at Dartmouth, 7400 Remsen, Hanover, NH 03755 USA; 2 Department of Systems Pharmacology & Translational Therapeutics, Perelman School of Medicine, University of Pennsylvania, Philadelphia, PA 19104 USA; 3 Division of Rheumatology, Department of Medicine, Boston University School of Medicine, Boston, MA USA; 4 Pulmonary Center, Department of Medicine, Boston University School of Medicine, Boston, MA 02118 USA; 5 Division of Rheumatology and Clinical Immunology, Department of Medicine, University of Pittsburgh Medical Center, Pittsburgh, PA 15261 USA; 6 Division of Medicine, University College London, London, UK; 7 Division of Rheumatology, Department of Medicine, Feinberg School of Medicine, Northwestern University, Chicago, IL 60611 USA; 8 Department of Microbiology and Immunology, Geisel School of Medicine at Dartmouth, Lebanon, NH 03756 USA; 9 Department of Neurological Sciences, Larner College of Medicine, University of Vermont, HSRF 426, 149 Beaumont Avenue, Burlington, VT 05405 USA

**Keywords:** Systemic sclerosis, Scleroderma, Macrophage, Lung disease, Functional genomics

## Abstract

**Background:**

Systemic sclerosis (SSc) is a multi-organ autoimmune disease characterized by skin fibrosis. Internal organ involvement is heterogeneous. It is unknown whether disease mechanisms are common across all involved affected tissues or if each manifestation has a distinct underlying pathology.

**Methods:**

We used consensus clustering to compare gene expression profiles of biopsies from four SSc-affected tissues (skin, lung, esophagus, and peripheral blood) from patients with SSc, and the related conditions pulmonary fibrosis (PF) and pulmonary arterial hypertension, and derived a consensus disease-associate signature across all tissues. We used this signature to query tissue-specific functional genomic networks. We performed novel network analyses to contrast the skin and lung microenvironments and to assess the functional role of the inflammatory and fibrotic genes in each organ. Lastly, we tested the expression of macrophage activation state-associated gene sets for enrichment in skin and lung using a Wilcoxon rank sum test.

**Results:**

We identified a common pathogenic gene expression signature—an immune–fibrotic axis—indicative of pro-fibrotic macrophages (MØs) in multiple tissues (skin, lung, esophagus, and peripheral blood mononuclear cells) affected by SSc. While the co-expression of these genes is common to all tissues, the functional consequences of this upregulation differ by organ. We used this disease-associated signature to query tissue-specific functional genomic networks to identify common and tissue-specific pathologies of SSc and related conditions. In contrast to skin, in the lung-specific functional network we identify a distinct lung-resident MØ signature associated with lipid stimulation and alternative activation. In keeping with our network results, we find distinct MØ alternative activation transcriptional programs in SSc-associated PF lung and in the skin of patients with an “inflammatory” SSc gene expression signature.

**Conclusions:**

Our results suggest that the innate immune system is central to SSc disease processes but that subtle distinctions exist between tissues. Our approach provides a framework for examining molecular signatures of disease in fibrosis and autoimmune diseases and for leveraging publicly available data to understand common and tissue-specific disease processes in complex human diseases.

**Electronic supplementary material:**

The online version of this article (doi:10.1186/s13073-017-0417-1) contains supplementary material, which is available to authorized users.

## Background

Integrative genomics has yielded powerful tissue-specific functional networks that model the interaction of genes in these specialized “microenvironments” [1]. These tools hold promise for understanding how genes may contribute to human diseases [2] that arise, in part, out of an aberrant interplay of cell types and tissues. Network biology has played a crucial role in our understanding of complex human diseases such as cancer [3, 4] and, more recently, in disorders where the interactions among multiple tissues are dysregulated [5]. Analytical approaches that leverage biological “big data” can be especially fruitful in rare and heterogeneous diseases [6], for which the risk of mortality is significant and no approved treatments exist. We performed an integrative, multi-tissue analysis for systemic sclerosis (SSc; scleroderma), a disease for which all of these tenets are true, and included samples from patients with pulmonary fibrosis (PF) and pulmonary arterial hypertension (PAH).

SSc is a systemic disease characterized by abnormal vasculature, adaptive immune dysfunction (autoantibody production), and extracellular matrix (ECM) deposition in skin and internal organs. The etiology of SSc is unknown, but it has complex genetic risk [7] and postulated triggers include immune activation by cancer [8], infection [9], or dysbiosis [10]. SSc is clinically heterogeneous, with some patients experiencing rapidly progressive skin and internal organ disease while others have stable disease that is largely limited to skin. Understanding the molecular processes in multiple affected organ systems is critical to understanding the pathogenesis of SSc and other complications, such as PF and PAH, that co-occur in these patients. Here, we ask if deregulated pathways are distinct or common between these tissues affected by SSc and if each organ manifestation has distinct disease signatures at the molecular level.

An integrative genomics study of SSc is of particular importance. Gene expression data from multiple tissues, including skin [11–13], lung [14, 15], and esophagus (ESO) [16], now exist. However, the rarity of the disease results in studies with small sample sizes and the multi-organ nature makes it difficult to assess molecular changes across organ systems relative to controls. Therefore, analyzing the data from multiple tissues that are more difficult to obtain (e.g., esophagus and lung) in the context of tissues that are more easily assayed (e.g., skin and peripheral blood) is a powerful way to make inferences about pathogenesis in internal organs. In addition, putting SSc disease-specific findings in the context of tools built from biological big data is a way to bolster and refine our findings for this rare disease.

We previously developed mutual information consensus clustering (MICC) to identify gene expression that is conserved across multiple, disparate datasets [17]. Here we expanded MICC to perform an integrative, multi-tissue analysis of SSc and related fibrotic conditions. We included gene expression datasets from ten different cohorts representing four different affected tissues from patients with SSc. Following MICC, we used the Genome-scale Integrated Analysis of gene Networks in Tissues (GIANT) tissue-specific functional genomic networks [1] to identify gene–gene interactions among those expressed consistently across affected tissues. These big data approaches integrate individual experiments measuring hundreds of disease states and biological perturbations. Integration of these data holds promise for understanding how genes contribute to organ-specific manifestations of human diseases [2]. These GIANT networks are a detailed, genome-scale representation of the functional interactions between genes in different microenvironments.

We identified a pathogenic signature—a common “immune–fibrotic axis”—that is present in all tissues analyzed and is increased in the most severe disease complications, including PF and PAH. Using tissue-specific functional networks [1], we analyzed the nature of the immune–fibrotic axis to understand the gene–gene interactions that underlie fibrosis across organ systems. Using differential network analysis, we were able to identify skin- and lung-specific gene–gene interactions relevant to macrophage (MØ) plasticity and SSc pathophysiology. We now propose a model that implicates alternatively activated MØs as part of the immune–fibrotic axis that may drive fibrosis in multiple tissues.

## Methods

### Patients and datasets

Eight out of ten datasets included in this study were previously published (Table 1). All patients in these studies met the American College of Rheumatology definition for SSc [18]; Additional file 1 summarizes the patient information to which we had access on a per-array basis. A total of 573 samples from 321 subjects recruited at seven independent centers were analyzed. These data represent samples from four different affected tissues derived from seven different clinic centers in the US and Europe. Data include SSc and control skin from a University of California, San Francisco cohort [11], a Boston University cohort [12], and a Northwestern University cohort [13, 17]. Many patients in the skin cohorts provided lesional (forearm) and non-lesional (back) skin biopsies; a subset of patients in the Northwestern skin cohort provided biopsies longitudinally over time as part of a clinical trial for mycophenolate mofetil. Peripheral blood mononuclear cells (PBMC) samples from patients with and without SSc-associated PAH (SSc-PAH), patients with idiopathic PAH (IPAH), and healthy controls were included from a Boston University cohort [19] and a University of Colorado PAH cohort [20]. Lung data contained a cohort of late or end-stage patients that underwent lung transplant at the University of Pittsburgh [15] and a second cohort of open lung biopsies from early SSc-associated PF (SSc-PF) obtained in Brazil [14]. The lung biopsies included patients with SSc-PF, idiopathic PF (IPF), SSc-PAH, and idiopathic PAH (IPAH). Data on previously unpublished samples were also included in these analyses. These are two datasets of skin biopsies from patients with limited cutaneous SSc (LSSc) recruited from University College London (UCL)/Royal Free Hospital and Boston University Medical Center. Only data that were judged to be high quality were included in the analyses. To our knowledge, there was no overlap between the patient cohorts beyond five patients recruited at Northwestern that provided both skin and esophageal biopsies. We summarize all patient cohorts in Additional file 1. A more detailed description of the patient populations and criteria for inclusion can be found in the primary publications.

**Table 1:** Datasets included in this study

Dataset label	Tissue	Phenotypes of interest	References	GEO accession
Milano	Diffuse skin	Inflammatory subset, proliferative subset	Milano et al. [11]	GSE9285
Pendergrass	Diffuse skin	Inflammatory subset, proliferative subset	Pendergrass et al. [12]	GSE32413
Hinchcliff	Diffuse skin	Inflammatory subset, proliferative subset	Hinchcliff et al. [13] Mahoney et al. [17]	GSE45485, GSE59785
LSSc	Limited skin	NA	Present study	GSE76806
UCL	Limited skin	NA	Present study	GSE76807
Christmann	Lung	SSc-PF	Christmann et al. [14]	GSE76808
Bostwick	Lung	SSc-PF, IPF, IPAH, SSc-PAH	Hsu et al. [15]	GSE48149
ESO	Esophagus	Inflammatory subset, proliferative subset, SSc-PAH	Taroni et al. [16]	GSE68698
PBMC	PBMC	SSc-PAH	Pendergrass et al. [19]	GSE19617
Risbano	PBMC	IPAH, SSc-PAH	Risbano et al. [20]	GSE22356

*Abbreviations*: *ESO* Esophagus﻿,﻿ *GEO* Gene Expression Omnibus, *IPAH* idiopathic pulmonary arterial hypertension, *IPF* idiopathic pulmonary fibrosis, *PAH* pulmonary arterial hypertension, *PBMC* peripheral blood mononuclear cells, *PF* pulmonary fibrosis, *NA* not available

We used the patient disease label (e.g., PAH) as published in the original work for all of these sets. Below, we note some important characteristics (for the purposes of this work) of the included patient populations. As noted in the “Results” section, the two lung datasets contained patients with different histological patterns of lung disease. Some patients included in the PBMC dataset, including those with PAH, also had interstitial lung disease, though exclusion of these patients does not significantly change the interpretation as put forth in Pendergrass et al. [19]. As illustrated in Additional file 1, two datasets (ESO, LSSc) did not contain healthy control samples and three datasets (UCL, LSSc, and PBMC) were comprised entirely of LSSc patients.

### Microarray dataset processing

This work contains ten datasets on multiple microarray platforms. Agilent datasets (Pendergrass, PBMC, Milano, Hinchcliff, ESO, UCL, LSSc) used either Agilent Whole Human Genome (4x44K) Microarrays (G4112F) (Pendergrass, PBMC, Milano, Hinchcliff, ESO, UCL) or 8x60K (LSSc). Data were Log_2_-transformed and lowess normalized and filtered for probes with intensity twofold over local background in Cy3 or Cy5 channels. Data were multiplied by −1 to convert to Log_2_(Cy3/Cy5) ratios. Probes with >20% missing data were excluded. The Illumina dataset (Bostwick, HumanRef-8 v3.0 BeadChips) was processed using variance-stabilizing transformation xand robust spline normalization using the lumi R package. Dr. Christmann provided the raw data in the form of.CEL files. Dr. Feghali-Bostwick provided Illumina BeadSummary files. Affymetrix datasets (Risbano, HGU133plus2; Christmann, HGU133A_2) were processed using the Robust Multiarray Averaging (RMA) method as implemented in the affy R package. Batch bias was detected in the ESO dataset. To adjust these data, missing values were imputed via *k*-nearest neighbor algorithm using a GenePattern [21] module with default parameters and the data were adjusted using ComBat [22] run as a GenePattern module to eliminate the batch effect.

To compare datasets in our downstream analysis, duplicate genes must not be present in the dataset and must be summarized in some way. First, we annotated each probe with its Entrez gene ID. Agilent 4x44K arrays were annotated using the hgug4112a.db Bioconductor package. LSSc was annotated using UNC Microarray Database with annotations from the manufacturer. Probes annotated to lincRNAs (A19) were removed from the analysis. The Illumina dataset was annotated by converting the gene symbols (provided as part of the BeadSummary file) to Entrez IDs using the org.Hs.eg.db package. The Risbano PBMC dataset was annotated using the hgu133plus2.db package. The Christmann dataset was annotated using an annotation file from the manufacturer. Probes that did not map to any Entrez ID and probes that mapped to multiple Entrez IDs were removed in all cases. Probes that mapped to the same Entrez ID were collapsed to the gene mean using the aggregate function in R, followed by gene median centering.

### Clustering of microarray data and statistical tests for phenotype association

The collapsed datasets were used to find coherent coexpression modules. We used Weighted Gene Co-expression Network Analysis (WGCNA), a strong clustering method, which allows us to automatically detect the number of coexpression modules and remove outliers [23]. Each dataset was clustered using the blockwiseModules function in WGCNA R package using the signed network option and power = 12; all other parameters were set to default. WGCNA does not identify large, densely connected coexpression modules in random data [23] and although changing the soft-thresholding power ultimately changes the resulting modules, we and others find the resulting modules to be stable and concordant across parameter choices [23].

Using the WGCNA coexpression modules also reduces the dimensionality of the dataset, as it allows us to test for genes’ association with, or differential expression in, a particular pathophenotype of interest on the order of tens, rather than thousands, using the module eigengene. The module eigengene is the first principal component and represents the expression of all genes in a module and an idealized hub of the coexpression module. We used the moduleEigengenes function in the WGCNA R package to extract the eigengenes. A module was considered to be pathophenotype-associated if the module eigengene was significantly differentially expressed in or significantly correlated with a pathophenotype of interest. Only two-class categorical variables were considered using a Mann–Whitney U test (i.e., all pulmonary fibrosis and pulmonary arterial hypertension patients were grouped together regardless of underlying etiology). We used Spearman correlation for continuous values. *P* values were Bonferroni-corrected on a per-phenotype basis. See Additional files 2, 3, 4, 5, 6, 7, 8, and 9 for complete output of these analyses. In the main text, we discuss categorical pathophenotypes, as these were enriched at the consensus cluster level. We do find instances of coexpression modules that are associated with continuous pathophenotypes, such as pulmonary function test measurements, but these were not apparent at the consensus cluster level of abstraction.

### Module overlap network construction and community detection

The ten-partite “module overlap network” was constructed as in Mahoney et al. [17], where it was called the “information graph” due to its relationship to information theory. We describe the method here in brief and refer to Mahoney et al. [17] for motivating details. The modules from different datasets have no a priori relationship to each other. The module overlap network encodes the pairs of modules that significantly overlap. Specifically, for each pair of modules (C_i_ and C_j_) we compute an overlap score:

1 $$ {W}_{i j}=\frac{\left|{C}_i{\displaystyle \cap }{C}_j\right|}{N} log\frac{\left|{C}_i{\displaystyle \cap }{C}_j\right|}{\left|{C}_i\right|\left|{C}_j\right|} $$

where N is the total number of genes shared between the two datasets. The overlap scores can be positive, negative, or zero, indicating that the modules overlap more, less, or the same as expected at random, respectively. As shown in Mahoney et al. [17], the overlap scores can be naturally thresholded using information theory to yield a sparse network of significant overlaps—the module overlap network. We performed a permutation test to test the significance of the mutual information between a pair of partitions (datasets) and found that the true value of the mutual information of partitions was higher than all sampled values of the null distribution (permuted *p* = 0; see Additional file 10 for permutation test details and Additional file 11 for the results of this test). This is consistent with mutual information being implicitly computed relative to a null model.

The module overlap network is highly structured. For example, a module representing an inflammatory process in skin often significantly overlaps inflammatory modules in other tissues. Thus, the structure of the module overlap network corresponds to the biological processes that are common to multiple datasets. We can identify these processes by clustering the module overlap network itself. Community detection is a procedure used to identify clusters in networks. The type(s) of community detection we employed is based on the concept of modularity (see also Additional file 12; Glossary of terms used in this paper). Networks with high modularity have sets of nodes (here, coexpression modules) that are more densely connected within a set and more sparsely connected outside of that set [24]. Community detection methods based on this concept take into account the expected amount of edges within a set of nodes and detect the sets of nodes that are more densely connected than expected (communities) [25].

We used two methods of community detection. First, we used fast-greedy modularity maximization (implemented in Matlab) [24], which yielded large, diffuse communities. (The fast-greedy modularity optimization algorithm has a known bias for the size of communities it selects and is thought to find “low-resolution” clusters in some cases [25].) We call these “top-level” communities. We tested whether the modularity of the module overlap network as calculated by fast-greedy community detection was significant relative to a network where module labels are randomly permuted. This allowed us to assess whether the module overlap graph had significant community structure; the results were highly significant (permuted *p* = 0; see Additional file 10 for permutation test details).

Because the above algorithm is greedy, it only finds a local maximum for modularity. To find smaller, more densely connected sub-communities, we used spin-glass community detection (igraph R package implementation, max number of communities = 10, all other parameters were set to default) [26, 27]. This algorithm implements a stochastic algorithm to maximize the modularity function resulting in tighter clusters than the fast-greedy algorithm [27]. We call these “bottom-level” communities. The community/sub-community structure of the module overlap network demonstrates that there is a hierarchy of biological processes that are common across datasets, where large communities contain smaller ones. To display this hierarchical community structure, we first sorted by top-level community label, and then within each community we sorted by bottom-level label. The adjacency matrix of the module overlap network and its node attributes (including dataset of origin and community labels) are supplied in Additional files 13 and 14, respectively.

We also tested each top-level community in the module overlap network for enrichment of pathophenotype-associated modules for each phenotype of interest using a Fisher’s exact test followed by Bonferroni correction (Table 2). This test takes into account both modules that had increased and decreased in pathophenotypes under study.

**Table 2:** Bonferroni-corrected *p* values, Fisher’s exact test pathophenotype-associated modules in top-level communities in the module overlap graph

Top-level community	“In SSc” *p* value	“In inflammatory” *p* value	“In proliferative” *p* value	“In PAH” *p* value	“In PF” *p* value
1	1	0.02	1	1	1
2	0.71	0.07	1	1	1
3	0.09	0.27	1	0.77	0.29
4	8.56E-07	6.30E-12	1	0.30	1
5	1	1	0.03	1	1
6	1	1	1	1	1
7	1	0.64	1	0.03	1
8	1	1	1	1	1

### Functional and pathophenotype annotation of the module overlap network

The module overlap network contains rich information about the biological processes that are active in each tissue under study. We functionally annotated the module overlap network by finding pathways that strongly correlate to each community. Because an edge in the module overlap network corresponds to a significant overlap between coexpression modules from different datasets, we can think of an edge “encoding” that overlap as a gene set. For each pair of coexpression modules *C*_*i*_ and *C*_*j*_, we define an “edge gene set”, *E*_*ij*_, as the overlap between the two datasets:

2 $$ {E}_{i j}={C}_i{\displaystyle \cap }{C}_j $$

To annotate this edge gene set with biological pathways, we computed the Jaccard similarity of an edge gene set *E* and a pathway *P*:

3 $$ J\left( E, P\right) = \frac{\left| E{\displaystyle \cap } P\right|}{\left| E{\displaystyle \cup } P\right|} $$

We used biological pathways from the Kyoto Encyclopedia of Genes and Genomes (KEGG) [28], BioCarta, and Reactome [29] obtained from Molecular Signatures Database from the Broad Institute (http://software.broadinstitute.org/gsea/msigdb). The Jaccard similarity between the edge and pathway will be equal to one if all of the genes shared between two modules are exactly the same set of genes annotated to the pathway, or zero if no genes are shared between the two sets. To functionally annotate a community in the information graph, we compared the Jaccard similarities of the edges within the community to edges outside of the community using a Mann–Whitney U test (with Bonferroni adjustment). The full results of this analysis are included as Additional files 15, 16, 17, 18, 19, 20, 21, 22, and 23.

### Tissue consensus gene sets

To understand how the immune and fibrotic responses in these phenotypes are functionally related, we found the consensus genes in the combined 4A and 4B clusters. Tissue consensus gene sets were derived by considering all modules within 4A and 4B, finding their unions within their dataset, and then computing their intersection across datasets from the same tissue of origin. For example, the lung consensus gene set (CC_lung_) was derived by computing the union of the Christmann (denoted *c*) and Bostwick (denoted *b*) modules in 4AB separately, and then computing the intersection across these two datasets:

4 $$ C{C}_{lung}=\left({\cup_{c\in C}}_{{}_{4 AB}} c\right){\displaystyle \cap}\left({\cup}_{b\in {B}_{4 AB}} b\right) $$

As each tissue was considered separately (limited skin and diffuse skin were considered separately), five tissue consensus gene sets were generated; the union of these tissue consensus datasets was used to query the functional genomic networks and is referred to as the “immune–fibrotic axis consensus” gene set or genes throughout the text. For all genes in modules in clusters 4A and 4B, we calculated the Pearson correlation to their respective module eigengene. We compared this correlation of consensus genes to that of non-consensus genes using a Mann–Whitney U test. Additional file 24 contains the tissue consensus genes from 4AB or the immune–fibrotic axis consensus genes.

### Querying GIANT functional networks, single tissue network analysis, and network visualization

The GIANT functional genomic networks were obtained as binary (.dab) files and processed using the Sleipnir library for computational functional genomics [30]. We queried all networks (lung, skin, “all tissue”, macrophage) using the immune–fibrotic axis consensus gene sets (as Entrez IDs) and pruned all low probability (<0.5) edges. All networks are available for download from the GIANT webserver (http://giant.princeton.edu/) [1]. For the single tissue analysis (e.g., lung network), we considered only the largest connected component of each network and performed spin-glass community detection as implemented in the igraph R package [27] to obtain the functional modules. We annotated functional modules using g:Profiler [31] using all genes in a module as a query. All networks in this work were visualized using Gephi [32]. The network layout was determined by community membership, the strength of connections between communities, and finally the interactions between individual genes. The lung network node attribute file and edge lists are supplied as Additional files 25 and 26.

### Differential network analysis

The tissue-specific networks from GIANT allow for the analysis of the differing functional connectivity between genes in different microenvironments. In order to understand the specific immune–fibrotic connectivity in lung relative to skin, we performed a differential network analysis. To compare networks we retained only nodes common to the largest connected components of the consensus skin and lung networks (see "Querying GIANT functional networks, single tissue network analysis, and network visualization"). We define the “differential lung network” as the network with adjacency matrix:

5 $$ {A}_{diff}= \max \left({A}_{lung}- \max \left({A}_{skin},\ {A}_{global}\right),0\right) $$

where A_lung_, A_skin_, and A_global_ are the lung, skin, and global (all tissues) adjacency matrices from GIANT. The differential lung network is thus the lung network minus the maximum edge weight from the skin and lung networks, where all edges that are stronger in skin or the global network are set to zero. Thus, the differential lung network contains only highly lung-specific interactions. Functional modules in the lung differential network were found using spin-glass community detection (see "Querying GIANT functional networks, single tissue network analysis, and network visualization") within the largest connected component of the network. The differential lung network node attributes and edge list are supplied as Additional files 27 and 28.

To perform the macrophage-specific network analysis in the supplemental material, we subtracted global edge weights from the macrophage network, setting negative edges to zero (as above). We then permuted the order of the adjacency matrix (edges) 1000 times and assessed if the true weight within a community was more than random (red), less than random (blue), or no different from random (white). We performed the same permutation testing on the lung network with global subtraction and found more weight than expected “on-diagonal” and less weight than expected “off-diagonal”; this demonstrates how spin-glass community detection takes into account the expected distribution of edges.

### Differential expression and MØ gene set analysis

To identify genes that were differentially expressed in SSc-PF, SSc-PF samples were compared to normal controls in both datasets using Significance Analysis of Microarrays (SAM [33]; 1000 permutations, implemented in the samr R package [33]). Genes with a false discovery rate (FDR) <5% were considered further. The MØ gene sets used in this study are WGCNA modules taken from a study of human MØ transcriptomes [34]. The z-score of each genes’ expression (Eq. 6) was computed in the collapsed Christmann and Hinchcliff datasets (as described in the “Microarray dataset processing” section of “Methods”). The z-score *z* of gene *g* in the *i*th array/sample is computed as:

6 $$ {Z}_{g i}=\frac{x_{g i}-{\mu}_g}{\sigma_g} $$

where *x*_*gi*_ is the gene expression value in array/sample *i*, *μ*_*g*_ is the gene mean, and *σ*_*g*_ is the gene standard deviation. The average z-score of genes in a set (module from Xue et al. [34]). computed for an array/sample to summarize gene set expression. Mann–Whitney U tests were used to compare average z-scores between groups. To validate these findings in an independent SSc skin dataset, we used the data from Assassi et al. [35] as processed by the authors and deposited in NCBI Gene Expression Omnibus (GEO; GSE58095 series matrix). We collapsed duplicated probes to the gene mean, z-scored genes as in Eq. 6, and compared average z-scores as above.

## Results

We performed an integrative analysis of ten independent gene expression datasets containing samples from patients with SSc and associated co-morbidities (Table 1). The primary goal of this study was to identify the fundamental processes that occur across end-target and peripheral tissues of patients with SSc and related fibrotic conditions. Secondly, we aimed to identify the presence or absence of common gene expression patterns that underlie the molecular intrinsic subsets of SSc [11] in different organs. Analysis of multiple tissue biopsies from patients with skin fibrosis, esophageal dysfunction, PF, and PAH allowed us to determine in a data-driven manner whether these tissues were perturbed in a similar manner on a genomic scale.

**Fig. 1:**
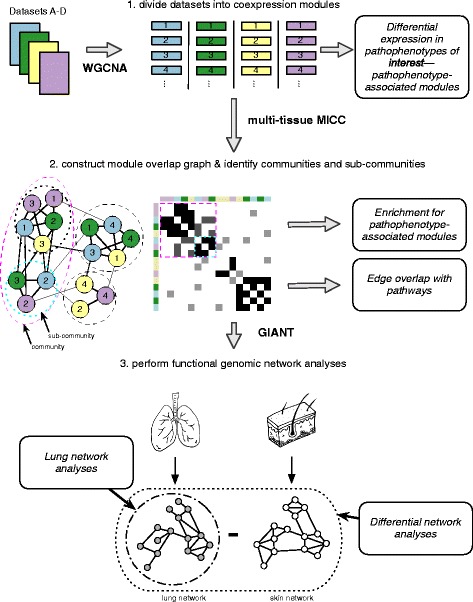
Schematic overview of the analysis pipeline. Four datasets are shown for simplicity. Each gene expression dataset was partitioned using WGCNA independently to obtain coexpression modules. Module eigengenes were tested for their differential expression in pathophenotypes of interest. Modules were compared across datasets using MICC to form the “module overlap graph” and community detection algorithms were used to identify communities and sub-communities in the graph. These communities correspond to molecular processes that are conserved across datasets. Each community was examined for enrichment of pathophenotype-associated modules and edge overlap with canonical biological pathways. Gene sets derived from these communities were used to query GIANT functional genomic networks. The resulting networks allow for tissue-specific interrogations of the gene sets. Differential network analysis was performed to compare the lung and skin networks

We applied MICC [17] to identify conserved, differentially co-expressed genes across all tissues in our SSc compendium. MICC is a “consensus clustering” procedure, meaning that it identifies the *shared co-clustering of genes* present in multiple datasets. MICC identifies genes that are consistently coexpressed in multiple tissues. Procedurally, MICC clusters gene expression data into coexpression modules using WGCNA (Fig. 1). Because this clustering is purely data-driven, coexpression modules derived from different datasets necessarily differ from each other. MICC integrates these coexpression modules across datasets by identifying significant overlaps between modules from different datasets and forming a “module overlap network”. MICC then parses the module overlap network to find sets of modules (communities) that are strongly conserved across many datasets (see “Methods”). These strongly overlapping modules correspond to molecular processes that are conserved across multiple datasets.

**Fig. 2:**
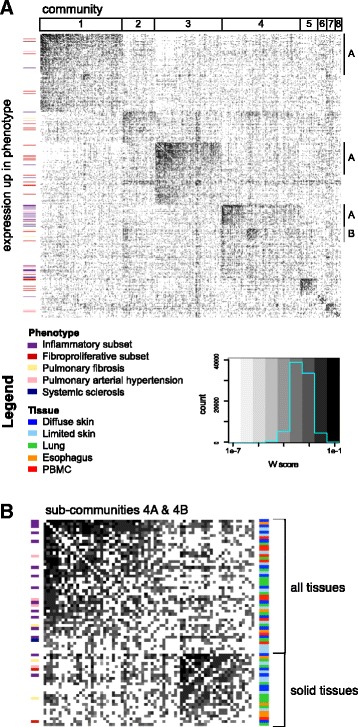
The multi-tissue module overlap graph demonstrates that severe pathophenotypes have similar underlying expression patterns. **a** The full adjacency matrix of the module overlap graph sorted to reveal hierarchical community structure. A *darker cell color* is indicative of a higher W score or larger edge weight. Communities (*numbered*) and sub-communities (*lettered*) are indicated by the *annotation tracks above* and on the *right side* of the matrix, respectively. Coexpression modules with expression that is increased in a phenotype of interest are marked by the *annotation bar* on the *left side* of the matrix. If a module was up in SSc as well as another pathophenotype of interest, the other pathophenotype color is displayed. **b** The adjacency matrix of sub-communities 4A and 4B indicates that these clusters contain modules that are up in all pathophenotypes of interest and show that there are many edges between the two sub-communities. Sub-community 4A contains modules from all tissues whereas 4B contains mostly solid tissue modules as indicated by the *tissue annotation track* to the *left* of the matrix

All datasets were partitioned into coexpression modules using WGCNA, resulting in 549 modules (Table 3). We constructed the ten-partite module overlap network (Fig. 2) and identified eight communities in the network using modularity-based community detection methods. Because the community structure of the module overlap network was hierarchical, we used a hierarchical labeling scheme, where numerals denote large communities and letters denote smaller sub-communities (Fig. 2a). For each community, we used set theoretic formulae to derive a final gene set (“consensus genes”) associated with the modules in that community (see “Methods”; Additional file 29; consensus gene sets ranged from 64–9597 genes in size). The majority of the consensus gene sets pertain to biological processes that are not necessarily disease-specific (e.g., there is no enrichment for genes [modules] that are differentially expressed in disease versus control in that community). These include processes such as telomere organization (1A) and macromolecule localization (3A). *Disease-specific* consensus genes were identified by first determining which communities were enriched for modules associated with pathophenotypes (e.g., contain differentially expressed genes in disease) under study and then deriving consensus gene sets from those combined communities (see "Severe pathophenotypes share a common immune–fibrotic axis").

**Table 3:** Number of microarrays and WGCNA coexpression modules in each of the datasets included in this study

Dataset	Number of arrays	Number of coexpression modules
Milano	75	39
Pendergrass	89	38
Hinchcliff	165	62
LSSc	24	39
UCL	15	98
Christmann	18	56
Bostwick	62	54
ESO	33	71
PBMC	54	38
Risbano	38	54

### Severe pathophenotypes share a common immune–fibrotic axis

The module overlap network is agnostic to the clinical phenotypes corresponding to each biopsy. To associate communities in the module overlap network with SSc and fibrotic pathophenotypes, we tested each of the 549 modules for differential expression in relevant pathophenotypes (see “Methods”). For example, every lung module in the PAH cohorts was tested for differential expression in PAH. Clusters 4A and 4B in the module overlap network contain modules with increased expression in all pathophenotypes of interest: the inflammatory and proliferative subsets of SSc, PAH, and PF (Fig. 2b). Thus, the modules in these communities correspond to a common, broad disease signal that is present in every pathophenotype under study. As with our prior studies, we did not find a strong association with autoantibody subtype and the co-expression modules identified here.

Edges in the module overlap graph represent overlap between coexpression modules in different datasets, so we identified the intersection of genes between adjacent modules. We then asked if these “edge gene sets” were similar to known biological processes by computing the Jaccard similarity between edges and canonical pathways from the Molecular Signatures Database [36]. Edges in 4A encode immune processes such as antigen processing and presentation and cytotoxic T-cell and helper T-cell pathways (Table 4). This cluster also contains modules from all tissues, including PBMCs (Fig. 2b). Altered immunophenotypes have been reported in SSc-PAH and SSc-PF [14, 19]. Here, we find that the immune processes with increased expression in these severe pathophenotypes have substantial overlap with each other, as well as with the inflammatory subsets in esophagus and skin (Fig. 2b; Additional file 30: Figure S1). Notably, 4A is composed of modules with increased expression in PAH in PBMCs and lung, and a module upregulated in end-stage PF (Additional file 30: Figure S1). This demonstrates a commonality of molecular pathways between the inflammatory component of SSc and the most severe end-organ complications at the expression level.

**Table 4:** Selected pathways that are similar to overlapping coexpression patterns in consensus clusters in the information graph

Consensus cluster	Summary of selected pathways
1A	DNA repair Cell cycle RNA metabolism Transcription
2	Cell–cell junction organization Aquaporin-mediated transport Tight junctions
3A	Endocytosis mRNA processing Metabolism of proteins
4A	T cytotoxic and helper pathway Antigen processing and presentation Allograft rejection
4B	ECM receptor interaction Collagen formation ECM organization TGF-beta signaling Signaling by PDGF
5	G2 M checkpoint Unwinding of DNA Cell cycle
6	Notch signaling Nuclear receptors in lipid metabolism and toxicity
7	Steroid biosynthesis Fatty acid metabolism PPAR signaling pathway
8	Keratin metabolism FGFR ligand binding and activation

We calculated the Jaccard similarity index between edges in the information graph and canonical pathways and used a Mann–Whitney U test to assess whether a particular pathway was more similar to edges within a consensus cluster than outside the consensus cluster

Edges in 4B encode pro-fibrotic processes, including ECM receptor interaction, collagen formation, and TGF-β signaling (Table 4). Cluster 4B consists of skin inflammatory and fibroproliferative subset-associated modules as well as lung PAH-, late PF- and early PF-associated modules (Fig. 2b; Additional file 30: Figure S1). These results replicate and expand what we have found in our prior meta-analysis of skin data alone [17]: the expression patterns observed in the SSc intrinsic subsets are *shared* with other tissues and SSc-associated pathophenotypes and indicative of altered immune and fibrotic processes (an immune–fibrotic axis).

To understand how the immune–fibrotic axis and these phenotypes are functionally related, we identified the consensus genes in the combined 4A and 4B clusters (see “Methods”; 2079 unique genes; Additional file 24). Consensus genes are highly central within their respective dataset gene–gene correlation networks and our procedure identifies sets of genes that capture disease-specific variation (Additional file 30: Figure S2). Using a conservative measure, these consensus genes are enriched for genes with increased expression in all disease manifestations (SAM [33], FDR <5%; PF in both lung datasets *p* < 2.2e-16; PAH lung, *p* = 7.88 × 10^−5^; PAH in both PBMC datasets, *p* = 3.20 × 10^−15^, Fisher’s exact test). This demonstrates that the tissue consensus genes are highly relevant to all disease manifestations in this study. The tissue consensus gene sets allow us to rigorously extrapolate from this conservative set a substantially broader, disease-associated signal. This extrapolation is especially important for tissue studies that are underpowered to detect a large number of significantly differentially expressed genes (see “Discussion”). We took the union of the tissue consensus gene sets as a set of “immune–fibrotic axis consensus genes” that are informative about pathology in every tissue.

### The lung functional genomic network reveals a coupling of immune and fibrotic processes

**Fig. 3:**
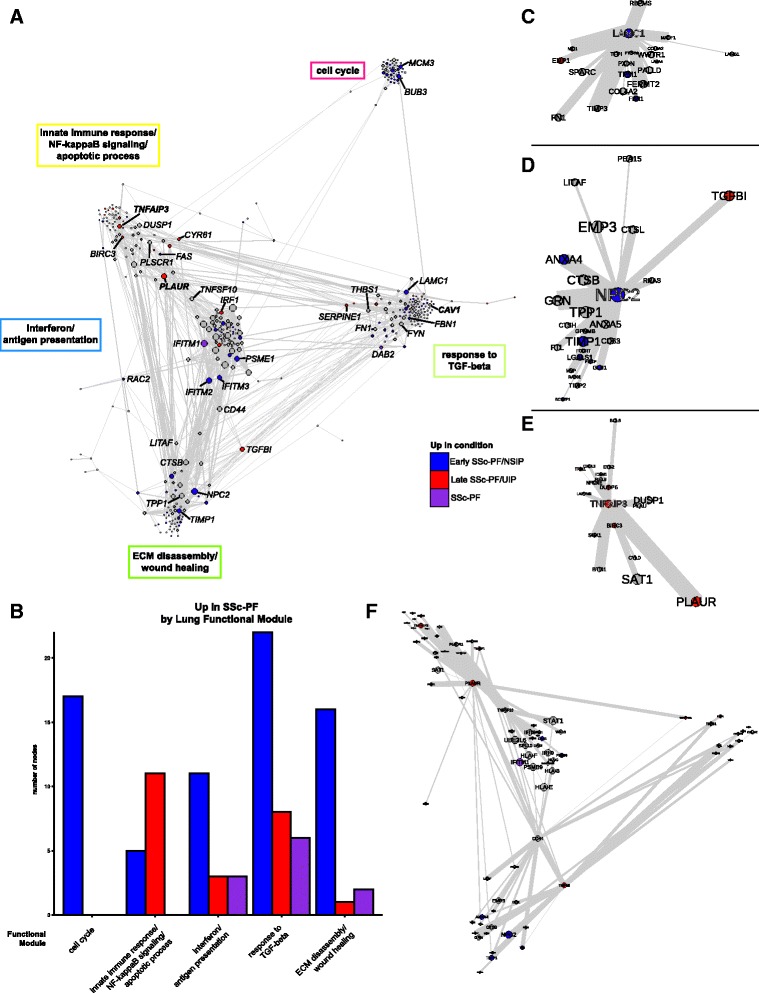
Genes that are overexpressed in late and early SSc-PF are distributed throughout the lung network. **a** The lung network shows functional connections between inflammatory and fibrotic processes. Genes in the largest connected component were clustered into functional modules using community detection. Biological processes associated with the functional modules are in *boxes* next to the modules. Genes are colored by whether they are overexpressed in *late SSc-PF/UIP* (*red*), *early SSc-PF/NSIP* (*blue*), both (*SSc-PF*, *purple*), or neither (*grey*). *NSIP* non-specific interstitial pneumonia, *UIP* usual interstitial pneumonia. Gene symbols in *bold* have putative SSc risk polymorphisms. Node (gene) size is determined by degree (number of functional interactions) and edge width is determined by the weight (probability of interaction between pairs of genes). The layout is determined by community membership, the strength of connections between communities, and finally the interactions between individual genes in the network. A fully labeled network is supplied as Additional file 30: Figure S3 and is intended to be viewed digitally. **b** Quantification of differentially expressed genes in each of the five largest functional modules. **c**–**e** Hubs of the consensus lung network; only the first neighbors of the hub that are in the same functional module are shown. **c** *LAMC1* is a hub of the response to TGF-beta module. **d** *NPC2* is a hub of the ECM disassembly, wound healing module. **e** *TNFAIP3* is a hub of the innate immune response, NF-κB signaling, and apoptotic processes module. **f** Bridges of the consensus lung network. First neighbors of *PLAUR*, *CD44*, *TNFSF10*, and *TGFBI* are shown

The GIANT functional networks infer functional relationships between genes by integrating publicly available data, including genome-wide human expression experiments, physical and genetic interaction data, and phenotype and disease data [1]. In these networks, genes are nodes and edges are weighted by the estimated probability of a tissue-specific relationship between genes. GIANT contains networks for multiple tissues, including skin and lung. To investigate the function of the immune–fibrotic axis consensus genes in pulmonary manifestations of SSc, we extracted the subnetwork of the GIANT whole genome lung network corresponding to the immune–fibrotic axis consensus genes—the *lung network* (Fig. 3; Additional file 30: Figure S3). Similar to our previous analysis of SSc skin, we find interconnected functional modules related to both immune (interferon (IFN)/antigen presentation and innate immune/NF-κB/apoptotic processes) and fibrotic (response to TGF-β and ECM disassembly/wound healing) processes (Fig. 3a). This demonstrates that, like skin, there is functional coupling between inflammatory and pro-fibrotic pathways in lung.

Our analysis includes two lung datasets derived from both early SSc-PF (open lung biopsies obtained for diagnostic purposes [14]) and end-stage or late disease (SSc-PF patients that underwent lung transplantation [15]). In addition to the differences in disease stage between these two datasets, there is also some difference in the histological patterns of fibrosis in these cohorts. In the Bostwick lung dataset [15], all patients with SSc-PF had usual interstitial pneumonia (UIP). This study used lung tissues from patients who underwent lung transplantation (late disease). The Christmann lung dataset [14] contains five patients with non-specific interstitial pneumonia (NSIP) and two patients with centrilobular fibrosis. This study looked at early SSc-PF patients, used open lung biopsies, and specifically avoided honeycombing areas.

Although NSIP and UIP have distinct clinical outcomes, they have been shown to be nearly indistinguishable at the gene expression level [37]. Furthermore, these datasets have overlapping coexpression patterns as demonstrated by their shared community membership in the module overlap network. Comparison of different datasets allows us to determine how genes with increased expression at these different stages and histological subtypes of lung disease are distributed throughout the lung network. Genes overexpressed in SSc-PF (SAM, PF versus Normal comparison, FDR <5%) are distributed throughout the lung network and therefore are predicted to participate in all of the molecular processes identified in the network. Quantification of the distribution of SSc-PF differentially expressed genes throughout the consensus lung network (Fig. 3b) demonstrates that molecular processes can be associated with either a disease stage, histopathological pattern, or both stages/patterns. The cell cycle module contains only early/NSIP SSc-PF genes, the innate immune response/NF-κB/apoptotic processes module contains more late/UIP SSc-PF genes, and the response to TGF-β module contains genes from *both* histological patterns (Fig. 3a, b).

### Hub and bridge genes are associated with the pathogenesis of pulmonary fibrosis

Certain genes occupy privileged positions within molecular networks and these genes often have critical biological function [38]. *Module hub genes* are connected to a significant fraction of genes within a functional module, whereas *bridge genes* are genes that connect to multiple functional modules and thus “bridge” them. We identified the hub and bridge genes within the lung network for their possible roles in PF pathogenesis. We highlight the hubs and bridges of the lung network in Fig. 3c–e and f, respectively. The hubs of several of the functional modules in the consensus lung network show increased expression at different disease stages or histological patterns (Fig. 3c–e). For instance, *LAMC1* shows increased expression in early/NSIP SSc-PF and is highly connected within the response to TGF-β module (Fig 3c). The gene Niemann-Pick disease, type C2 (*NPC2*) is upregulated in early disease and is connected to cathepsins L and B (*CTSL*, *CTSB*) and *GLB1* in the lung network (Fig 3d). We tabulate information on selected genes from the lung network in Table 5.

**Table 5:** Selected genes in the consensus lung network

Functional module	Gene symbol	Description	Network position	Up in	Function/potential role in disease
Cell cycle	*BUB3*	BUB3 mitotic checkpoint protein	-	Early SSc-PF/NSIP	Encodes a mitotic cell cycle checkpoint protein that regulates the onset of anaphase
	*CDC7*	Cell division cycle 7	-	-	Regulates MCM complex
	*MCM3*	Minichromosome maintenance complex component 3	-	Early SSc-PF/NSIP	Subunit of minichromosome maintenance (MCM) complex
	*MSH6*	MutS homolog 6	-	Early SSc-PF/NSIP	Participates in DNA mismatch repair.
ECM disassembly/wound healing	*CD44*	CD44 molecule (Indian blood group)	Bridge	-	A hyaluronic acid receptor that can interact with many other ligands found in the ECM. Primary idiopathic PF fibroblasts exhibit an invasive phenotype that was abrogated with treatment with anti-CD44 [39]
	*CD63*	CD63 molecule	-	-	Has been observed to interact with TIMP1 [83]
	*CTSB*	Cathepsin B	-	-	Regulates NPC2 secretion, TNF-alpha production, and cholesterol trafficking genes in an animal model of obesity [51]
	*CTSL*	Cathepsin L	-	-	Regulates NPC2 secretion, TNF-alpha production, and cholesterol trafficking genes in an animal model of obesity [51]
	*GLB1*	Galactosidase, beta 1	-	Early SSc-PF/NSIP	Mutations in this gene can lead to GM1-gangliosidosis, a manifestation of which includes foam cell accumulation in the lungs [84]
	*NPC2*	Niemann-Pick disease, type C2	Hub	Early SSc-PF/NSIP	Mutations in this gene result in a lipid storage disorder. Functions in the regulation of cholesterol trafficking through the lysosome by binding to cholesterol released from low density lipoproteins taken up by cells
	*TGFBI*	Transforming growth factor, beta-induced	Bridge	Late SSc-PF/UIP	Induced by phagocytosis of apoptotic debris in monocyte-derived MØs and regulates collagen turnover [44]
	*TIMP1*	TIMP metallopeptidase inhibitor 1	-	Early SSc-PF/NSIP	Has been observed to interact with CD63 and overexpression has been noted to inhibit apoptosis in a CD63-dependent manner [83]
Innate immune response/NFkB signaling/apoptotic process	*BIRC3*	Baculoviral IAP repeat-containing protein 3	-	Late SSc-PF/UIP	Has antiapoptotic activity through interactions with caspases as well as the TNF superfamily members TRAF1 and TRAF2 [85, 86]
	*CYR61*	Cysteine-rich, angiogenic inducer, 61		Late SSc-PF/UIP	Also known as CCN1. Implicated in apoptosis in fibroblasts [87]. Has been shown to play a role in Fas-mediated and TRAIL-induced apoptosis [88, 89]
	*DUSP6*	Dual specificity phosphatase 6	-	Late SSc-PF/UIP	Plays a role in the positive regulation of apoptosis [90]
	*FAS*	Fas cell surface death receptor	-	Early SSc-PF/NSIP	Cell surface death receptor
	*NFKBIE*	Nuclear factor of kappa light polypeptide gene enhancer in B-cells inhibitor, epsilon	-	-	Negative regulator of NFkB signaling
	*PLAUR*	Plasminogen activator, urokinase receptor	Bridge	Late SSc-PF/UIP	Also known as uPAR. Contains an SSc risk SNP. Pulmonary fibroblasts from patients with idiopathic PF over express uPAR and that uPAR ligation results in a hypermotile phenotype [40]
	*PLSCR1*	Phospholipid scramblase 1	-	-	Regulates phospholipid membrane asymmetry
	*TNFAIP3*	Tumor necrosis factor, alpha-induced protein 3	Hub		Also known as A20. Contains an SSc risk SNP (also associated with other autoimmune conditions). Negative regulator of NFkB signaling
	*TNFSF10*	Tumor necrosis factor (ligand) superfamily, member 10	Bridge	-	Also known as TRAIL. Elevated in serum of SSc patients [91]
	*TNFRSF10B*	Tumor necrosis factor receptor superfamily, member 10b	-	Late SSc-PF/UIP	Also known as TRAILR2
IFN/antigen presentation	*HLA-E*	Major histocompatibility complex, class I, E	-	-	Class I MHC molecule
	*HLA-F*	Major histocompatibility complex, class I, F	-	-	Class I MHC molecule
	*IFITM1*	IFN induced transmembrane protein 1	-	SSc-PF (UIP and NSIP)	IFN signaling
	*IFITM2*	IFN induced transmembrane protein 2	-	Early SSc-PF/NSIP	IFN signaling
	*IFITM3*	IFN induced transmembrane protein 3	-	Early SSc-PF/NSIP	IFN signaling
	*IRF1*	IFN regulatory factor 1	-	Late SSc-PF/UIP	Activator of type I IFN signaling
	*OAS1*	2′-5′-Oligoadenylate synthetase 1, 40/46 kDa	-	Early SSc-PF/NSIP	Involved in innate immune response to viral infection
Response to TGF-beta	*CAV1*	Caveolin 1	-	-	Contains an SSc risk SNP
	*CTGF*	Connective tissue growth factor	-	-	Also known as CCN2. Has been shown to play a role in Fas-mediated and TRAIL-induced apoptosis [88, 89]
	*DAB2*	Dab, mitogen-responsive phosphoprotein, homolog 2 (Drosophila)	-	SSc-PF (UIP and NSIP)	Required for the epithelial to mesenchymal transition induced by TGF-beta in mouse and for type II TGFbR recycling [92, 93]
	*FN1*	Fibronectin 1	-	-	Extracellular matrix protein.
	*LAMC1*	Laminin gamma1 chain	Hub	Early SSc-PF/NSIP	Expression of this gene is essential for the development of basement membranes [94]
	*THBS1*	Thrombospondin 1	-	-	Mediates cell-to-cell and cell-to-matrix interactions. Putative biomarker of modified Rodnan skin score [95]

The innate immune response/NF-κB signaling/apoptotic process module contains genes that are highly expressed in late/UIP SSc-PF, including the hub genes *CYR61* and *TM4SF1* (Fig. 3a, b; Additional file 30: Figure S3). The hub gene *TNFAIP3* (A20), which is increased in late SSc-PF (Fig. 3e), is a negative regulator of NF-κB signaling and inhibitor of TNF-mediated apoptosis. The innate immune response/NF-κB signaling/apoptotic process and IFN/antigen presentation modules are bridged by *TNFSF10*, also known as TRAIL (TNF-related apoptosis inducing ligand; Fig. 3f). These results suggest that the balance of apoptosis is altered in late/UIP SSc-PF. The upregulation of genes with anti-apoptotic function was not reported in the original study [15], which demonstrates the strength of both the MICC method and the study of functional interactions.

*CD44* and *PLAUR* (uPAR) bridge multiple functional modules in the lung network (Fig. 3f) and have been implicated in IPF [39, 40]. Because these genes link modules important in regulating disease progression, therapeutic targeting of CD44 and uPAR may be an effective strategy in combating SSc-PF. Indeed, anti-CD44 treatment reduces fibroblast invasion and bleomycin-induced lung fibrosis [39], and inhibition of uPAR ligation significantly reduces motility of pulmonary fibroblasts from patients with idiopathic PF [40]. These results are consistent with our identification of these genes as key genes in the lung network.

### The lung microenvironment provides a distinct milieu for pro-fibrotic processes

**Fig. 4:**
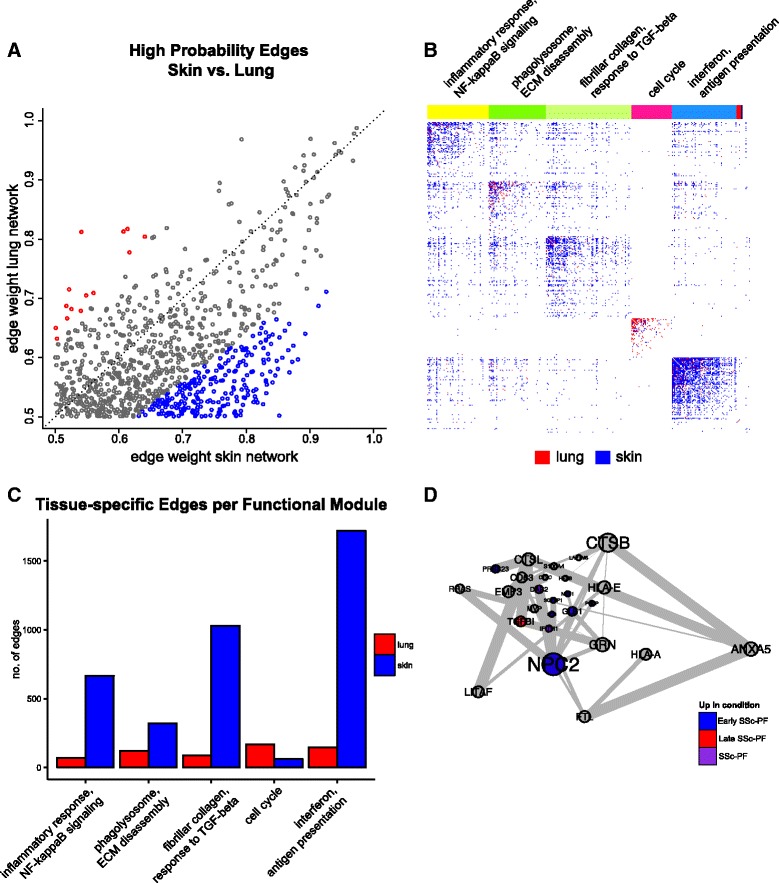
The lung and skin network structures indicate distinct tissue microenvironments influence fibrosis. The skin and lung networks were compared by first finding the giant component of the lung network and then collapsing to nodes only found in both the skin and lung networks (which are termed the common skin and common lung networks). **a** A scatterplot of high probability edges (>0.5 in both networks) illustrates that pairs of genes with a higher probability of interacting in skin than lung exist and vice versa. Edges are colored *red* if the weight (probability) is 1.25 times higher in lung or *blue* if it is 1.25 times higher in skin. **b** The differential adjacency matrix where a cell is colored if the edge weight in a given tissue is over and above the weight in the global average and tissue comparator networks. For instance, a cell is *red* if the edge weight was positive following the successive subtraction of the global average weight and skin weight. Community detection was performed on the common lung network to identify functional modules; common functional modules largely recapitulate modules from the full lung network. Representative processes that modules are annotated to are above the adjacency matrix. The annotation track indicates a gene’s functional module membership. Nodes (genes) are ordered within their community by common lung within community degree. A fully labeled heatmap is supplied as Additional file 30: Figure S4 and is intended to be viewed digitally. **c** Quantification of tissue-specific interactions in each of the five largest functional modules. **d** The lung-resident MØ module found in the differential lung network (consists only of edges in *red* in **b**)

Pulmonary fibrosis is histologically distinct from skin fibrosis and occurs in a subset of patients with SSc. We hypothesized that the lung microenvironment may have a distinct organization of immune–fibrotic axis consensus genes when compared to skin. Indeed, for interactions (edge weight >0.5) that are present in both the lung and skin networks, there are gene pairs that are much more likely to interact in one tissue than the other (Fig. 4a). In other words, the skin and lung networks are “wired differently”. To identify *highly lung-specific* and *highly skin-specific interactions*, we performed a differential network analysis that identified gene pairs that are strongly predicted to interact in one tissue but not the other (see “Methods”).

These highly specific interactions are displayed in Fig. 4b, where a cell is red if it is lung-specific or blue if it is skin-specific (cf. Additional file 30: Figure S4). The number of tissue-specific edges in each functional module is quantified in Fig. 4b, c, which illustrate that most functional modules in lung have fewer interactions than in skin, with the exception of the cell cycle module. Of particular interest is the relationship between the phagolysosome/ECM disassembly genes and response to TGF-β genes, as strong differential connectivity can be observed in this module (Fig. 4b, c). Thus, even though ECM disassembly and TGF-β module genes are coordinately differentially expressed in both lung and skin, they are differentially connected to each other, suggesting that the microenvironment strongly determines the functional consequences of upregulating these pro-fibrotic genes.

To summarize lung-specific biological processes in the immune–fibrotic axis, we clustered the lung-specific interactions (differential lung network) to identify lung-specific pathways (Additional file 30: Figure S5). We identified 23 clusters corresponding to biological processes such as type I IFN signaling (cluster 10), antigen processing and presentation (cluster 4), REACTOME cell surface interactions at the vascular wall (cluster 22), and mitotic cell cycle (cluster 16; shown in Additional file 30: Figure S5b). Taken together, this suggests that within the immune–fibrotic axis we find innate immune and cell proliferation processes that are highly lung-specific. One of the largest of these clusters (cluster 13; Fig. 4d; Additional file 30: Figure S5c) includes *NPC2*, *S100A4*, and *CTSB*, which encode protein products that are highly expressed in normal lung-resident MØs (LR-MØs) [41, 42].

*NPC2* is a hub of the ECM disassembly/wound healing module in the full lung network (Fig. 3); many of the genes in cluster 13 also belong to the ECM disassembly/wound healing module in the whole network, including the cathepsins *CTSB* and *CTSL*. Alveolar MØs are the main source of cathepsins in bleomycin-induced fibrotic lung tissue [43]. Additional genes associated with development and maintenance of alternative MØ activation include *TGFBI* [44], *NEU1* [45], *PRCP* [46], and *DAB2* [47]. Genes that are specifically associated with alternative activation of lung MØs include *PLP2* [48] and *IFITM1* [49] (Fig. 4d; Additional file 30: Figure S5c). Based on these genes and the complete lung network in Fig. 3, we identified an LR-MØ signature. These findings are consistent with previous reports of alternative MØ activation in SSc [14, 50].

**Fig. 5:**
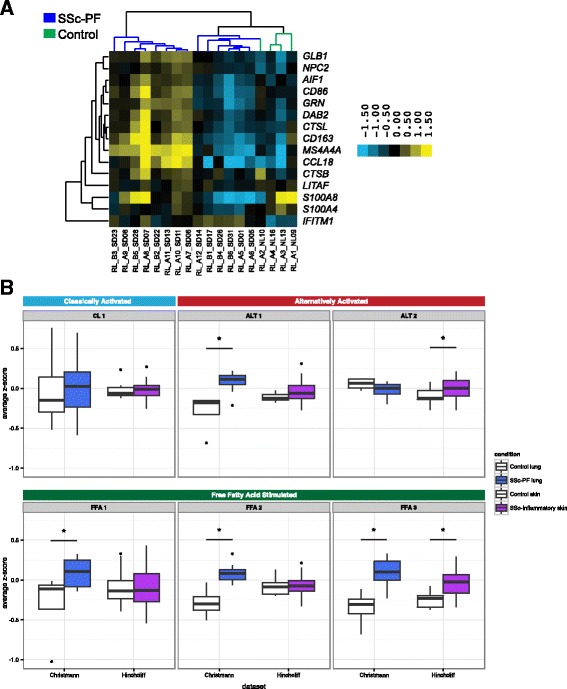
Evidence for alternative activation of MØs in SSc-PF lung that is distinct from SSc skin. **a** Genes identified by differential network analysis and inferred to be indicative of lung-resident MØs are correlated with canonical markers of alternatively activated MØs such as *CCL18* and *CD163* in the Christmann dataset. **b** Summarized expression values (mean standardized expression value) of gene sets (coexpression modules) upregulated in various MØ states from the Christmann and Hinchcliff datasets: module CL1, classic activation (IFN-γ); modules ALT 1 and 2, alternative activation (IL-4, IL-13); modules FFA 1, 2, and 3, treatment with free fatty acids. *FFA* free fatty acid. Modules from [34]. Asterisks (*) indicate significant differences (*p *< 0.05﻿﻿)

To explore this signature further, we examined some genes from this cluster along with genes identified in the Christmann et al. study [14]. Consistent with the primary publication [14], some heterogeneity in SSc-PF gene expression is observed and is likely due to tissue sampling from various lobes of the lung as well as the inclusion of patients with centrilobular fibrosis (Fig. 5a, right dendrogram branch). Nevertheless, the LR-MØ signature comprises genes that are highly correlated with canonical markers of alternatively activated MØs that were validated by either PCR or immunohistochemistry in the original study (e.g., *CD163* and CCL18). We also observed that genes in the phagolysosome/ECM disassembly functional module identified in lung are more highly connected in a macrophage-specific network than is expected at random (Additional file 30: Figure S6).

The LR-MØ cluster in the differential lung network also contains a number of genes implicated in lipid storage disorders, including *HEXB*, *GLB1*, and *NPC2*. Several other LR-MØ cluster genes have been shown to be important for regulating cholesterol trafficking genes in an animal model of obesity, including *CTSB*, *CTSL*, and *NPC2* [51]. It has been noted that lipid metabolism genes are upregulated in lung MØs relative to other tissue-specific MØs [48]. Furthermore, in the bleomycin injury mouse model of pulmonary fibrosis, lipid-laden MØs have been observed to increase expression of markers associated with alternative MØ activation and to secrete TGF-β [52].

### Distinct MØ gene expression programs are elevated in lung and skin

We hypothesized that early SSc-PF lung samples may have evidence of both alternatively activated and lipid-stimulated MØs and that this may differ from what is observed in skin. The presence of alternatively activated MØs in the inflammatory subset of skin was inferred in our single tissue analysis [17]. To test this hypothesis, we used gene sets associated with classic activation of MØs, alternative activation of MØs, or stimulation of MØs with a variety of activation stimuli, including free fatty acids, taken from Xue et al. [34]. To summarize the expression of each MØ gene set [34] and compare across tissues in these data, we computed the average expression of all genes in each gene set (see “Methods”; see Additional file 31 for a mapping between Xue et al. modules and our naming scheme). Results are displayed for control and SSc-PF lung, as well as control and SSc-inflammatory skin (Fig. 5b). As shown in Fig. 5b, there is evidence of an increase in alternatively activated and free fatty acid stimulated gene sets in SSc-PF and SSc-inflammatory skin. These data do not show statistically significant differences in expression of gene sets associated with classic MØ activation between controls and SSc-PF or SSc-inflammatory skin (see Additional file 32 for *p* values of all modules tested).

The discovery of IFN-related genes among the consensus genes indicates that these pathways are increased in pathophenotypes of interest (e.g., SSc-PF and the skin inflammatory subset). Christmann et al. also noted a strong IFN-related gene signature in SSc-PF samples, although the cellular compartment responsible for this signature was not described [14]. Because stimulation with IFN results in classic activation of MØs, we examined the expression of genes from CL 1, as it is most strongly associated with IFN-γ treatment (“classic activation”) in human MØs [34]. However, CL 1 genes’ expression is not different between disease and controls in either skin or lung (Wilcoxon *p* = 0.76 and 0.80, respectively; Fig. 5b). This result is consistent with our inability to discern differences in classic MØ activation markers between controls and SSc-PF and inflammatory skin and suggests that classically activated MØs are not the source of the reported IFN signature we find.

Modules ALT 1 and ALT 2 are both associated with IL-4 and IL-13 treatment, which are stimuli associated with alternative activation of MØs [34]. These two gene sets are non-overlapping coexpression modules and therefore represent two “parts” of the alternatively activated MØ transcriptional program. We performed functional enrichment analysis for ALT 1 and 2 to understand which biological processes underlie these transcriptional signatures (see “Methods”). Module ALT 1 is enriched for genes involved in oxidative phosphorylation (KEGG, *p* < 0.0001) and the citric acid cycle (REACTOME, *p* < 0.0001) pathways. In lung, ALT 1 expression is higher in SSc-PF than in controls (Wilcoxon *p* = 0.0046). There is no difference between healthy controls and the inflammatory subset in skin (Wilcoxon *p* = 0.41). Module ALT 2 shows an opposite trend and is enriched for genes implicated in the positive regulation of response to wounding (Gene Ontology (GO) biological process (BP), *p* = 0.027) and defense response (GO BP, *p* = 0.00035); this module includes alternatively activated MØ markers such as *CD14* and *CCL26* [53, 54]. ALT 2 expression is increased in the inflammatory subset in skin (Wilcoxon *p* = 0.041) and trends toward decreased expression in SSc-PF lung (Wilcoxon *p* = 0.16). Together, these pathways suggest a metabolic “switch” associated with alternative activation in lung that is not found in skin (for review see [55]; Fig. 5b).

We also analyzed modules associated with free fatty acid (FFA) stimulation, which are relevant to the question of lipid signaling or exposure in SSc tissues (FFA 1, 2, and 3). We first performed functional enrichment analysis for these modules to gain biological insight into these transcriptional programs. FFA 1 is enriched for genes involved in the unfolded protein response (REACTOME, *p* = 0.025). FFA 2 is enriched for antigen processing-cross presentation genes (REACTOME; *p* = 0.00101). FFA 3 is enriched for genes in the ER-phagosome pathway (REACTOME, *p* = 0.0076). Expression of FFA 1 and 2 is significantly increased in lung (FFA 1 Wilcoxon *p* = 0.046, *p* = 0.97 in skin; FFA 2 Wilcoxon *p* = 0.0013, *p* = 0.63 in skin), whereas FFA 3 is upregulated in SSc-PF lung (Wilcoxon *p* = 0.0013) and the SSc inflammatory subset in skin (Wilcoxon *p* = 0.00056). These results suggest that LR- MØs may have a distinct lipid exposure that strongly diverges from that in skin.

We repeated this analysis in an independent SSc skin dataset (Assassi et al. [35]) to validate our findings (Additional file 30: Figure S7). Assassi et al. reported that macrophage transcripts are elevated in SSc skin but used a “general” to macrophages signature gene list that does not provide information about activation state. The results from Assassi et al. largely agree with the results from the Hinchcliff dataset: ALT 2, but not ALT1 (Wilcoxon *p* = 0.0682), is significantly increased in SSc skin (Wilcoxon *p* = 5.92e-05), and FFA 3 is the only FFA module significantly increased in SSc skin (Wilcoxon *p* = 3.219e-06; FFA 1 *p* = 0.928; FFA 2 *p* = 0.486). The only disparity between the two skin datasets is that we find that CL 1 is significantly increased in the Assassi SSc patients (*p* = 0.000856). This difference may be because we looked at all SSc patients rather than “fibroinflammatory” patients alone, or due to the increased coverage of the genome on the platform used. Overall, analysis of Assassi et al. supports the differences in MØ alternative activation programs and lipid response in SSc-affected skin and lung.

The differential network analysis (Fig. 4) allowed us to identify highly lung-specific interactions in the immune–fibrotic axis that implicated lipid signaling as a distinct functional process in lung. The higher expression of *multiple* free fatty acid-associated modules in lung suggests that the role of lipid signaling in MØs may be more important in this tissue than in skin, consistent with what we would predict based on *highly lung-specific* gene–gene interactions, and based on prior biomedical literature in related conditions [48, 52]. Thus, a major difference between the lung and skin networks can be attributed to the presence of a distinct MØ phenotype in lungs.

## Discussion

SSc is a systemic disease that affects multiple internal organs. Herein, we present the first study of molecular mechanism of disease across multiple affected organ systems in SSc. To our knowledge, we show for the first time that a common set of cell types and pathways are driving disease across these affected organs, and importantly that it can also be found in related fibrotic conditions.

Gene expression data have been collected for multiple tissues in SSc and related conditions. However, these data often have issues that are common to many rare diseases. First, SSc is not prevalent and patients with particular disease manifestations are still rarer, so there is a limit to the amount of biopsy material available for study. Second, for practical and ethical reasons, internal organ biopsies are seldom taken from healthy subjects, making comparisons difficult. Thus, lung, esophagus, and other affected internal organs are more difficult to study than blood and skin tissue. Therefore, there is a critical need to leverage our biological prior knowledge with our understanding of well-studied tissues—like blood and skin—to make plausible inferences about pathogenesis in tissues that are more difficult to study.

The clinical heterogeneity of SSc, particularly the difficulty of predicting internal organ involvement, raises an important question: are the fibrotic processes observed in multiple organs derived from a common disease process, or is each organ manifestation effectively a distinct disease? Our analyses demonstrate that there is a common gene expression signature underlying all severe organ manifestations of SSc—the immune–fibrotic axis—in solid organs. The immune–fibrotic axis underlies both SSc pulmonary manifestations of PF and PAH, and the intrinsic subsets of skin and esophagus. Moreover, coexpression modules from peripheral blood, a mixture of innate and adaptive immune cells, have significant overlap with modules associated with all pathophenotypes studied. Thus, while fibrotic processes were largely associated with solid tissues, the inflammatory component of the immune–fibrotic axis is only found in peripheral blood.

The presence of a common gene expression signature across multiple tissues suggests a common disease driver, but it does not resolve the possible tissue-specific processes that contribute to disease in the internal organs. Indeed, there are many layers of biological regulation between gene expression and whole tissue phenotypes. Resolving the relationship between molecular profiles and phenotypes is a difficult biological problem underlying most biomedical inquiry. However, these relationships have been approximated by integrating high-throughput genomic data into tissue-specific functional networks using big data machine-learning strategies [1]. We addressed tissue specificity in SSc pathology by interpreting the common expression signal—the immune–fibrotic axis—within these tissue-specific functional networks. These networks allowed us to identify critical genes that occupy important positions in molecular pathways in lung. It is clear from this work that the coupling of immune and fibrotic processes is a hallmark of SSc that occurs in SSc-PF and SSc-PAH as well as skin. However, we also find subtle, lung-specific functional differences that we attribute, in part, to the plasticity of the myeloid cell lineage.

### The plasticity of the myeloid lineage may drive tissue-specific SSc disease processes

Altered immune function has been implicated in the pathogenesis of SSc [56, 57]. In most prior studies, characterization of macrophage activation has relied on analysis of a very limited number of surface markers and/or a few characteristic mRNAs [56, 57]. Most of these studies have concluded that SSc macrophages bear an M2 activation profile based on CD163 and/or CD206 expression. Macrophage polarization spans a broad spectrum of activation states, ranging from “classically activated” or M1 cells, which largely mediate pro-inflammatory responses to “alternatively activated” or M2 cells, which are predominantly associated with immune suppression and wound healing. While expression of CD206 and CD163 is higher in alternatively activated macrophages compared with “classically activated” macrophages, it is difficult to make global generalizations about macrophage activation based on such limited analysis. While operationally useful, the designation of M1 versus M2 activation has limited utility in vivo as macrophage activation is informed by the local cytokine milieu to which these cells are exposed.

Our study of multiple skin cohorts showed that multiple gene expression markers of activated MØs are elevated in SSc skin across multiple data sets, consistent with gene expression profiling of lung tissue from SSc patients with interstitial lung disease [14]. These data are consistent with elevated levels of IL-4 and IL-13 in SSc sera [58, 59]. Furthermore, CD68^+^ MØs have been identified as producers of IL-13 in human SSc skin biopsies and genetic deficiency of IL-13 is protective against disease in a mouse model of SSc [60]. IL-13 activates tissue fibrosis [61] and genetic and observational studies link IL-13 with SSc pathogenesis [62–64].We have further demonstrated that SSc MØs express high levels of profibrotic cytokines, suggesting they play a significant role in mediating fibrosis and in maintaining an inflammatory environment in SSc (unpublished data).

By performing a combined analysis of SSc gene expression in multiple tissues, we are able to observe and infer, in a genome-wide manner, commonalities in the complex mixture of cell types in a tissue at the time of biopsy. Overwhelmingly, we detected a MØ signature associated with severe disease. In the module overlap network, we find that PAH-associated modules from PBMCs [19, 20] have significant overlap with SSc inflammatory subset-associated modules from skin and esophagus (Fig. 2). Indeed, in Pendergrass et al. [19], we observed that PBMCs from lcSSc patients have significant enrichment in myeloid- and MØ-related gene sets compared to healthy controls. Christmann et al. [65] expanded on this, showing that highly expressed transcripts in LSSc-PAH CD14^+^ monocytes were induced in IL-13-stimulated cells, i.e., that PAH monocytes are alternatively activated. We assert that this MØ polarization is a significant part of the immune–fibrotic axis we find in these data and, therefore, is likely *a common driver* of the complex pathophysiology of SSc. In support of this, an independent study also identified MØs and dendritic cells (DCs) as possible sources of an “inflammatory” signature in lesional SSc skin [35].

We found evidence for the contribution of LR-MØs to SSc-PF pathobiology, consistent with the alternative activation of MØs and TGF-β production. In our prior analysis of skin, we inferred alternatively activated MØs as modulators of the SSc inflammatory intrinsic subset in skin [17]. Our current study identifies a LR-MØ signature within the functional relationships of immune–fibrotic axis consensus genes in lung (Figs. 4d and 5a). We posit that the differences in fibrotic responses of skin and lung tissue are due, in large part, to innate differences between tissue-resident MØs that have been observed [66, 67], as well as the interactions between infiltrating monocytes and tissue-resident cell types (e.g., alveolar epithelial cells versus keratinocytes). Because MØ phenotype and function are plastic and readily modulated by the local tissue microenvironment, it is likely that differential activation of MØs in these tissues is the result of exposure to distinct cytokine milieu. Indeed, we show that distinct alternative activation gene expression programs have increased expression in SSc-PF lung and inflammatory SSc skin (Fig. 5). In particular, there were multiple lipid-related signatures elevated in SSc-PF lung alone.

We cannot rule out that the MØ changes we observe are a secondary response to the affected organ pathology. Regardless, therapies that target MØ effectors such as IL6R have shown promise in clinical trials [68] and MØ chemoattractants have been shown to be important in animal models of SSc inflammatory disease, suggesting that MØs play a central role in SSc pathogenesis. We also cannot rule out that DCs contribute to our results, as plasmacytoid DCs are observed to be important in the stiff skin syndrome mouse model [69]. However, some skin-resident DCs have been shown to be transcriptionally similar to peripheral blood monocytes in humans [70]. We speculate that the circulation of peripheral myeloid cells contributes to the multi-organ nature of SSc. Future studies may use in silico and cell-sorting techniques to deconvolve SSc expression data to identify changes in cell proportion and transcriptome throughout the disease course and to finely phenotype myeloid cells from SSc patient tissue samples.

### Summary of SSc-PF disease processes

**Fig. 6:**
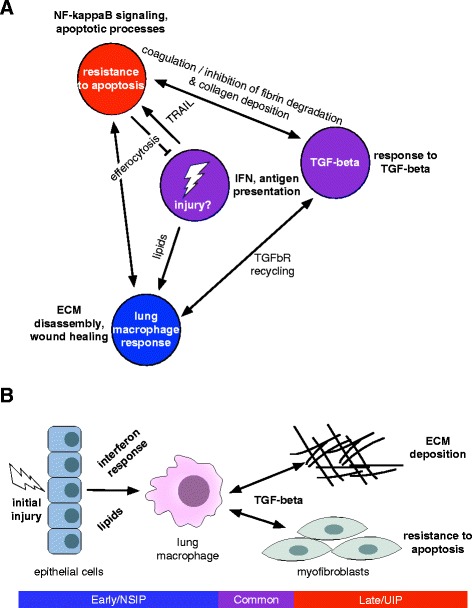
Overview of SSc-PF disease processes. **a** Network-centric overview. **b** Cell type-centric overview

The study of two different lung datasets that sampled early- and late-stage/UIP SSc-PF allows us to describe differences between the disease processes found in these two datasets. The two datasets each contained patients with different types of interstitial pneumonia (see “Methods”), which may limit interpretation of these results. However, as stated in the results, we and others [37] find evidence of highly similar gene expression patterns between UIP and NSIP. We do not have treatment information for patients in these studies and acknowledge that late-stage patients are more likely to be treated with immunosuppressive therapy. With these caveats in mind, we can nevertheless draw non-intuitive conclusions through the combination of our data-driven approach and mechanistic insight from disparate literature. We provide an overview of disease processes we observe in SSc in Fig. 6.

We found that gene signatures that are increased in alternatively activated human MØs and MØs treated with free fatty acids are enriched in early SSc-PF patients and that there is no evidence for enrichment of a pro-inflammatory, IFN-stimulated MØ signature (Fig. 5) [34].

Christmann et al. had previously identified an increase in IFN- and TGF-β-regulated genes in biopsies from early SSc-PF [14], but it was unclear which cell types were responsible for the IFN signature or if there was evidence of distinct subpopulations of MØs. Increased CCL18 protein and higher *CD163* mRNA were observed in lungs of patients with SSc-associated interstitial lung disease, suggestive of the presence of alternatively activated MØs [14].

We also find elevated gene expression programs associated with MØ alternative activation (specifically metabolic “reprogramming”) and lipid exposure in Christman et al. (Fig. 5).The LR-MØ signature identified in our differential network analysis consisted of genes with increased expression in early SSc-PF that participate in lipid and cholesterol trafficking (Fig. 4d; Additional file 30: Figure S5). The expression of these genes is correlated with “canonical” MØ genes identified in [14] (Fig. 5). In the bleomycin injury mouse model of pulmonary fibrosis, lipid-laden MØs, or foam cells, have been observed to upregulate markers associated with alternative MØ activation and to secrete TGF-β [52]. Oxidized phospholipid treatment also causes alternative activation and TGF-β secretion in human MØs [52]. Consistent with this report, recent work demonstrates that foam cell formation in vivo favors the development of a pro-fibrotic MØ activation profile [71, 72]. These studies, along with our results, suggest that lipid exposure or uptake in MØs may be important.

We find genes from both datasets in the response to the TGF-β module of the lung network. TGF-β signaling is a hallmark of SSc and other fibrotic diseases, and was noted in the initial analysis of both SSc lung datasets [14, 15]. However, we also find evidence that the type I IFN signature is present in the Bostwick dataset (Fig. 3). The functional module most strongly associated with late stage disease/UIP is the innate immune, NF-κB, and apoptotic processes module. This module is connected to the TGF-β module through components of the fibrinolysis pathway such as PAI-1 (*SERPINE1*; Fig. 3). PAI-1 is upregulated in late stage SSc-PF and is known to be important in pulmonary fibrosis [73–75]. One mechanism by which fibrinolysis may contribute to the resolution of fibrosis is through the induction of fibroblast apoptosis [76]. Both TGF-β1 and PAI-1 have been shown to inhibit lung fibroblast apoptosis [76].

We found evidence for a shift in the balance of apoptosis in the Bostwick dataset, perhaps in myofibroblasts [77], in our network analyses (Fig. 6). Long-lived myofibroblasts are thought to continually deposit collagen and contribute to persistent fibrosis [78]. This apoptotic-resistance phenotype is related to the stiffness of the matrix [79], suggesting that a shift in apoptotic processes may occur once the deposition of excess collagen begins. Moreover, impaired phagocytosis of apoptotic cells, or efferocytosis, has been observed in the alveolar MØs of IPF patients [80]. We find genes involved in efferocytosis, specifically in receptors (*CD44*) and endocytic machinery associated with this process, in the lung network (Figs. 3 and 6) [81]. If the shift in apoptosis and efferocytosis occurs, we speculate that the fibrotic and inflammatory processes in our network will also be altered. Efferocytosis by alveolar MØs plays a key role in the resolution of inflammation in the lung through the subsequent release of TGF-β [82]. We hypothesize that, following initial injury, TGF-β signaling, antifibrinolytic factors, and the disruption of apoptosis and efferocytosis may contribute to progressive fibrosis in SSc-PF (Fig. 6).

### Limitations and future directions

A limitation of this study is a lack of post-genomic validation, particularly in lung. This work is in essence hypothesis-generating, but the need for this study is highlighted by the sparseness of biopsy material, and it provides new directions for inquiry into the pathogenesis of the disease.

Our results suggest that alternatively activated MØs likely play a central role in the pathogenesis of SSc by activating fibroblasts. Most importantly, they show for the first time that this is likely to occur across multiple affected organ systems in SSc patients. Future experiments will need to examine these cells functionally to determine if SSc MØs can activate other cell types (e.g., fibroblasts) to produce ECM and to examine the role of these cells in mouse models of fibrosis as well as gene expression in multiple organs from the same patient. Our integrative genomics approach directly compares multiple tissues and manifestations and suggests that there may be subtle differences in the MØ phenotype in SSc-affected skin and lung. This supports the fine phenotyping of these cells from SSc patient tissue samples when possible, and the possibility of targeting these cells therapeutically.

## Conclusions

In this study, we have utilized data from multiple tissues to examine the systemic nature of SSc. Our integrative analysis allowed us to leverage well-studied tissues to inform us about SSc manifestations that are under-studied molecularly. This study rigorously tests the notion that patients with severe disease have shared immunological and fibrotic alterations. The common immune–fibrotic axis shows evidence for alternatively activated MØs in multiple SSc tissues. However, there are subtle differences in the MØ gene expression programs detected in skin and lung. Different microenvironments likely provide distinct stimuli to infiltrating MØs that determine the pro-fibrotic character of these cells. The plasticity of this lineage is likely central to the divergence of fibrotic processes in multiple SSc-affected tissues and is a central component of an immune–fibrotic axis driving disease.

## Additional files

Additional file 1: Table describing clinical characteristics of cohorts included in this study. (PDF 50 kb)

Additional file 2: Table of Bostwick dataset pathophenotype associations with WGCNA co-expression modules. (TXT 3 kb)

Additional file 3: Table of Christmann dataset pathophenotype associations with WGCNA co-expression modules. (TXT 2 kb)

Additional file 4: Table of ESO dataset pathophenotype associations with WGCNA co-expression modules. (TXT 4 kb)

Additional file 5: Table of Hinchcliff dataset pathophenotype associations with WGCNA co-expression modules. (TXT 4 kb)

Additional file 6: Table of Milano dataset pathophenotype associations with WGCNA co-expression modules. (TXT 3 kb)

Additional file 7: Table of PBMC dataset pathophenotype associations with WGCNA co-expression modules. (TXT 1 kb)

Additional file 8: Table of Pendergrass dataset pathophenotype associations with WGCNA co-expression modules. (TXT 2 kb)

Additional file 9: Table of Risbano dataset pathophenotype associations with WGCNA co-expression modules. (TXT 772 bytes)

Additional file 10: Permutation test supplementary methods and table detailing hypotheses and results. (PDF 74 kb)

Additional file 11: Table of z-scores from mutual information of partitions permutation test. (TXT 1 kb)

Additional file 12: Glossary of terms. (PDF 1303 kb)

Additional file 13: Module overlap graph adjacency matrix. (TXT 1262 kb)

Additional file 14: Table of coexpression module consensus cluster membership in module overlap graph. (TXT 7 kb)

Additional file 15: Table of consensus cluster 1 full output of edge-pathway (Jaccard) similarity Mann–Whitney U tests. (TXT 68 kb)

Additional file 16: Table of consensus cluster 2 full output of edge-pathway (Jaccard) similarity Mann–Whitney U tests. (TXT 60 kb)

Additional file 17: Table of consensus cluster 3A full output of edge-pathway (Jaccard) similarity Mann–Whitney U tests. (TXT 70 kb)

Additional file 18: Table of consensus cluster 4A full output of edge-pathway (Jaccard) similarity Mann–Whitney U tests. (TXT 61 kb)

Additional file 19: Table of consensus cluster 4B full output of edge-pathway (Jaccard) similarity Mann–Whitney U tests. (TXT 68 kb)

Additional file 20: Table of consensus cluster 5 full output of edge-pathway (Jaccard) similarity Mann–Whitney U tests. (TXT 68 kb)

Additional file 21: Table of consensus cluster 6 full output of edge-pathway (Jaccard) similarity Mann–Whitney U tests. (TXT 57 kb)

Additional file 22: Table of consensus cluster 7 full output of edge-pathway (Jaccard) similarity Mann–Whitney U tests. (TXT 68 kb)

Additional file 23: Table of consensus cluster 8 full output of edge-pathway (Jaccard) similarity Mann–Whitney U tests. (TXT 66 kb)

Additional file 24: Table of immune–fibrotic axis consensus genes. (TSV 27 kb)

Additional file 25: Node attribute file for lung network (Fig. 3). (TXT 17 kb)

Additional file 26: Edge list for lung network (Fig. 3). (TXT 27 kb)

Additional file 27: Node attribute file for differential lung network (Fig. 4). (TXT 4 kb)

Additional file 28: Edge list for differential lung network (Fig. 4). (TXT 7 kb)

Additional file 29: Table of consensus gene set sizes. (TSV 561 bytes)

Additional file 30: Supplementary figures and legends. (PDF 3174 kb)

Additional file 31: Table mapping Xue et al. module numbers to our module names (Fig. 5b). (TSV 125 bytes)

Additional file 32: Table of *p* values of all Xue et al. modules tested. (TSV 526 bytes)

## Abbreviations

DC: Dendritic cell
ECM: Extracellular matrix
FDR: False discovery rate
FFA: Free fatty acid
GEO: Gene Expression Omnibus
GIANT: Genome-scale Integrated Analysis of gene Networks in Tissues
IFN: Interferon
IPAH: Idiopathic pulmonary arterial hypertension
IPF: Idiopathic pulmonary fibrosis
LR-MØ: Lung resident macrophage
LSSc: Limited cutaneous systemic sclerosis
MICC: Mutual information consensus clustering
MØ: Macrophage
NSIP: Non-specific interstitial pneumonia
PAH: Pulmonary arterial hypertension
PBMC: Peripheral blood mononuclear cell
PF: Pulmonary fibrosis
SAM: Significance Analysis of Microarrays
SSc: Systemic sclerosis
SSc-PAH: scleroderma-associated pulmonary arterial hypertension
SSc-PF: scleroderma-associated pulmonary fibrosis
UCL: University College London
UIP: Usual interstitial pneumonia
WGCNA: Weighted Gene Co-expression Network Analysis
