# Supplementary material for: A novel multi-network approach reveals tissue-specific cellular modulators of fibrosis in systemic sclerosis
Source: Genome Med. 2017 Mar 23;9:27. doi: 10.1186/s13073-017-0417-1 (PMC5363043; doi:10.1186/s13073-017-0417-1)
Supplement: Supplementary file 1 — Table describing clinical characteristics of cohorts included in this study. (PDF 50 kb) [file 13073_2017_417_MOESM1_ESM.pdf]

Dataset	Pendergrass		Milano			Hinchcliff			Bostwick						Christmann		LSSc			UCL		ESO	PBMC			Risbano			
All subjects	SSc<br>N = 22	Control<br>N = 9	SSc<br>N = 24	Control<br>N = 6	Morphea<br>N = 3	SSc<br>N = 34	Control<br>N = 11	Morphea<br>N = 1	SSc<br>N = 14	SSc-PF<br>N = 8	SSc-PAH<br>N = 9	IPF<br>N = 13	IPAH<br>N = 8	Control<br>N = 9	SSc-PF<br>N = 7	Control<br>N = 4	SSc<br>N = 12	SSc-PAH<br>N = 8	SSc-ILD<br>N = 4	SSc<br>N = 10	Control<br>N = 5	SSc<br>N = 16	SSc<br>N = 21	SSc-PAH<br>N = 15	Control<br>N = 10	SSc<br>N = 10	SSc-PAH<br>N = 10	IPAH<br>N = 8	Control<br>N = 10
Age*, mean (SD)	46.1 (9.1)	NA	51.8 (10.4)	40.2 (10.6)	50.7 (2.9)	47.7 (11.7)	41.2 (11.8)	47	49.1 (9.2)	48.1 (7.5)	52.1 (9.1)	NA	NA	NA	44.7 (9.3)	NA	56.5 (12.8)	59 (13.4)	56.5 (17.8)	NA	NA	52.3 (8.7)	49.5 (10.9)	65.4 (6.4)	NA	NA	NA	NA	NA
Sex, n (% women)	17 (77.3)	NA	21 (87.5)	5 (83.3)	3 (100.0)	32 (94.1)	7 (63.6)	1 (100.0)	11 (78.6)	5 (62.5)	5 (55.6)	NA	NA	NA	NA	NA	9 (75.0)	7 (87.5)	4 (100.0)	NA	NA	15 (93.8)	17 (81.0)	12 (80.0)	3 (30.0)	NA	NA	NA	NA
SSc patients only	N = 22		N = 24			N = 34			N = 31						N = 7		N = 24			N = 10		N = 16	N = 36			N = 20			
SSc subtype, n (% diffuse)	22 (100.0)		17 (70.8)			31 (91.2)			14 (45.2)						2 (28.6)		0 (0.0)			0 (0.0)		10 (62.5)	0 (0.0)			NA			
SSc disease duration*, mean (SD)	18.4 (13.1) mos		7.7 (7.2) yrs			52.7 (67.4) mos			NA						74 (76.6) mos		NA			NA		105.0 (105.3) mos	NA			NA			
SSc autoantibody, n (% positive)																													
Scl-70	3 (13.6)		5 (20.8)			9 (26.5)			6 (19.4)						2 (28.6)		NA			NA		6 (37.5)	NA			NA			
RNA Pol III	3 (13.6)		NA			9 (26.5)			2 (6.5)						NA		NA			NA		6 (37.5)	NA			NA			
ACA	1 (4.5)		2 (8.3)			2 (5.9)			6 (19.4)						NA		NA			NA		NA	NA			NA			
SSc intrinsic subset, n (%)																													
Inflammatory	9 (40.9)		5 (20.8)			17 (50.0)			NA						NA		NA			NA		6 (37.5)	NA			NA			
Proliferative	8 (36.4)		11 (45.8)			7 (20.6)			NA						NA		NA			NA		4 (25.0)	NA			NA			
Normal-like	2 (9.1)		4 (16.7)			7 (20.6)			NA						NA		NA			NA		NA	NA			NA			
Limited	NA		3 (12.5)			2 (5.9)			NA						NA		NA			NA		NA	NA			NA			
* At base biopsy																													
