# Supplementary material for: A novel multi-network approach reveals tissue-specific cellular modulators of fibrosis in systemic sclerosis
Source: Genome Med. 2017 Mar 23;9:27. doi: 10.1186/s13073-017-0417-1 (PMC5363043; doi:10.1186/s13073-017-0417-1)
Supplement: Supplementary file 10 — Permutation test supplementary methods and table detailing hypotheses and results. (PDF 74 kb) [file 13073_2017_417_MOESM10_ESM.pdf]

Permutation test of mutual information

We used *mutual information of partitions* (MIP) to assess the amount of information that a module assignment (a partition) in one data set correlates with another assignment in another data set as described in Mahoney *et al*. [1]. To test the significance of the total mutual information between a pair of partitions, we performed the following permutation test. For each pair of partitions, we randomly permuted the module labels of one of the partitions, which preserves the number and size of modules while randomizing genes to modules. This produces a *null pair* of partitions where modules only overlap due to “random noise”. We then computed MIP for this null pair as a sample from the *null distribution*. Because data sets had different probe sets and WGCNA calls outliers that do not belong in any module, we computed MIP for genes that were present in both data sets and not outliers in either data set.

We performed the above randomization 100 times for each pair of data sets to compute a null distribution. In all cases, the true value of MIP was higher than all sampled values of the null distribution (empirical *p*-value = 0; see table below and Additional File 4). To quantify the difference between the true and null values, we computed the *z-score* of the true value as the number of standard deviations above the mean for the true value. The - *z*-scores were between 33.4 and 188.3, indicating that the true MIP values are well outside what is expected of random “jitter” in the module labels. This is expected, as MIP is explicitly scaled to the null model where module labels are uniformly random with respect to each other.

Permutation test of modularity in the module overlap network

We used modularity maximization to identify communities in the module overlap network. To test whether the module overlap network contained significant community structure, we tested whether the *modularity statistic* of the network was significantly higher than expected using the following permutation test. The module overlap network is a 10-partite network that encodes the significant overlaps among modules across data sets. For each pair of data sets, we randomly permuted the module identifiers in each data set to obtain a *null network*. This randomization preserves all of the mutual information and network structure between each pair of data sets, and the 10-partitite structure of the full network, but randomly breaks any structure that persists across multiple data sets. We recomputed the modularity statistic in the null network as a sample from the *null distribution*. We used the function ‘fastgreedy.community’ from the ‘iGraph’ R package [2] to calculate the modularity values.

We performed the above randomization 1000 times. The true modularity value was higher than all sampled values of the null distribution (empirical *p*-value = 0; see table below). The *z*-score of the true value (see above) was 121.3, indicating that the module overlap network has communities that are dramatically stronger than expected in null data. This demonstrates that the many modules identified in one data set have significant overlaps with modules in many other data sets.

**Analysis**	**Hypothesis**	**Robustness check**	***p*-value (*z*-score)**
WGCNA	Gene expression is clustered into significant modules that do not appear in random data.	Horvath *et al.* 2008 [3]	NA
Mutual information	Modules in different data sets overlap more than expected if modules labels were random.	Permutation test; randomly permute module labels 100 times.	All p = 0 (33.4 < z < 188.3) Additional File 11
Consensus clustering	The module overlap network contains significant communities that are absent in a random network with same edge weight distribution.	Permutation test on modularity statistic; randomly permute module identifiers within each bipartite component 1000 times.	p = 0 (z = 121.3)
Module association with pathophenotypes	Module expression is significantly correlated to pathophenotypes.	Wilcoxon rank sum test; Bonferroni corrected for multiple hypotheses	Additional Files 2-9
Association of consensus clusters with pathophenotypes	Pathophenotype-associated modules are enriched in particular communities in the module overlap network.	Fisher's exact test; Bonferroni corrected for multiple hypotheses	Table 2
Curated pathway (KEGG, Biocarta, REACTOME) enrichment in consensus clusters	Links in module overlap network communities are enriched for pathways relative to links outside the community.	Jaccard similarity measure & Wilcoxon rank sum test; corrected for multiple comparisons.	Additional Files 15-23

References

1. Mahoney JM, Taroni J, Martyanov V, Wood TA, Greene CS, Pioli PA, Hinchcliff ME, and Whitfield ML. Systems Level Analysis of Systemic Sclerosis Shows a Network of Immune and Profibrotic Pathways Connected with Genetic Polymorphisms. PLoS computational biology. 2015;11(1):e1004005.

2. Csardi G, and Nepusz T. The igraph software package for complex network research. InterJournal, Complex Systems. 2006;1695(5):1-9.

3. Horvath S, and Dong J. Geometric interpretation of gene coexpression network analysis. PLoS Comput Biol. 2008;4(8):e1000117.
