# Supplementary material for: A novel multi-network approach reveals tissue-specific cellular modulators of fibrosis in systemic sclerosis
Source: Genome Med. 2017 Mar 23;9:27. doi: 10.1186/s13073-017-0417-1 (PMC5363043; doi:10.1186/s13073-017-0417-1)
Supplement: Supplementary file 12 — Glossary of terms. (PDF 1303 kb) [file 13073_2017_417_MOESM12_ESM.pdf]

Glossary of terms used in this paper.

The analysis pipeline presented in the main text is motivated by the need to integrate several bioinformatics analyses across multiple datasets, tissues, and pathophenotypes (disease phenotypes). Here, we compile a set of terms that: 1) are used in a technical sense in mutual information consensus clustering (MICC), 2) may be standard in the bioinformatics/machine learning literature, or 3) are outside the scope of the main text. Many of these definitions are largely adapted from Mahoney, et al. (Mahoney et al, 2015)

Network/Graph

Networks are *any* collections of objects (called **nodes**) that have relationships with each other (called **edges**). The nature of the nodes and edges in a network determines the character of the network and what information it encodes. A standard, alternative term for a network is a graph. The terms network and graph are used interchangeably, but typically we use network when the nodes represent physically real things (like genes or proteins), whereas we use graph when the nodes are an abstraction of some sort.

There are 2 distinct types of networks that are used in the multi-tissue MICC method. They are given here in order of abstraction:

1) Gene-gene coexpression networks derived from a single gene expression data cohort: nodes are genes, edges are correlations between expression patterns.

2) The module overlap graph (Fig 2): nodes are modules derived from the gene-gene coexpression networks, links are similarity scores between modules derived from *different* datasets. An edge indicates that the two modules have a larger than expected overlap if the two gene-gene expression networks were completely dissimilar. Note that edges only connect modules from different datasets.

There is one additional type of network that is used in our current study, the Genome-scale Integrated Analysis of gene Networks in Tissues (GIANT) Bayesian functional genomic networks. The nodes of the GIANT networks are genes and the edges represent high probability functional interactions between those genes (e.g. the genes form a complex under some circumstance). These networks are learned from a large-scale compendium of publicly available gene expression data using tissue-specific gold standards. A functional genomic gene-gene network differs from a gene-gene coexpression network as the edges represent probabilities rather than correlations.

Node

Nodes are the basic unit of a network. They are the objects whose relationships are encoded in the network. For example, nodes could be genes and the network encodes some notion of relationship between genes.

Edge/Link

Edges are the unit of *relationship* between nodes in a network. A standard, alternative term for an edge is a link. Edges denote that a pair of nodes is related. Edges in our case are *weighted* meaning that they denote the strength of a relationship between nodes. For example, edges in a gene-gene network could represent the correlation of those genes in a particular experiment. The weight in this case is the strength of the correlation.

Cluster/Clustering

A cluster is any grouping of objects by a notion of similarity between objects. There are two notions of cluster used in MICC: gene expression clusters (coexpression modules) and consensus clusters. The former are sets of genes that are similar in the sense that they are coexpressed *within* a single gene expression dataset. The latter are sets of the gene expression modules that are similar in the sense that they a broadly conserved *across* datasets.

Clustering is any algorithmic procedure that identifies groups of similar objects.

WGCNA

Weighted gene coexpression network analysis (WGCNA) is a clustering procedure takes gene expression data from a single cohort and finds groups of genes that are highly correlated to each other and weakly correlated outside of their group. WGCNA is built upon the notion of a gene coexpression network (see above) and extracts a small set of signals that account for a large fraction of the gene expression variance.

Coexpression module

A coexpression module is an alternative term for a cluster of genes that are grouped together by coexpression. Module is the standard term applied the output of Weighted Gene Coexpression Network Analysis (WGCNA).

Module overlap graph

The module overlap graph is network whose nodes are coexpression modules from distinct datasets and edges represent a *significantly large overlap* between those modules.

Consensus cluster & consensus genes

A consensus cluster is a set of genes whose coexpression is preserved across multiple datasets. MICC is a procedure for *identifying* these sets of genes. Communities in the module overlap graph are said to be consensus clusters. We refer to genes in a consensus cluster as consensus genes.

> *Tissue consensus genes* – sets of genes identified by considering all coexpression modules in a consensus cluster in the module overlap graph, computing their union within a dataset and computing the intersection across datasets from the same tissue of origin. For example, the lung consensus gene set from a consensus cluster would be derived by

computing the union of the Christmann and Bostwick coexpression modules separately, and then computing the intersection across these two datasets.

"IFP consensus" or "disease consensus" genes – the union of tissue consensus gene sets from consensus clusters 4A and 4B in the multi-tissue module overlap graph.

Consensus network

A consensus network is the part of (subgraph) the GIANT functional network that is made up of or is connected to the SSc disease consensus genes. The consensus lung network is the network that results from querying the GIANT lung-specific functional genomic network with the SSc disease consensus genes.

Differential network

We use the term differential network—specifically, differential lung network—to refer to the *highly lung-specific* edges in the consensus lung network. These are the edges that remain after subtracting off the tissue-naïve ("global") and skin-specific functional genomic network edges.

Partition

A partition is a grouping of elements of a set into distinct groups. For example, WGCNA forms a partition of the genome by clustering genes into distinct, non-overlapping modules. The whole collection of modules from WGCNA comprises the partition.

Functional module/molecular module

In the context of a functional genomic network, we refer to a community as a functional module. We expect the subgroup of genes (nodes) in a community in a functional genomic network to participate in a coherent biological (functional) process based on their predicted interactions.

Adjacency matrix

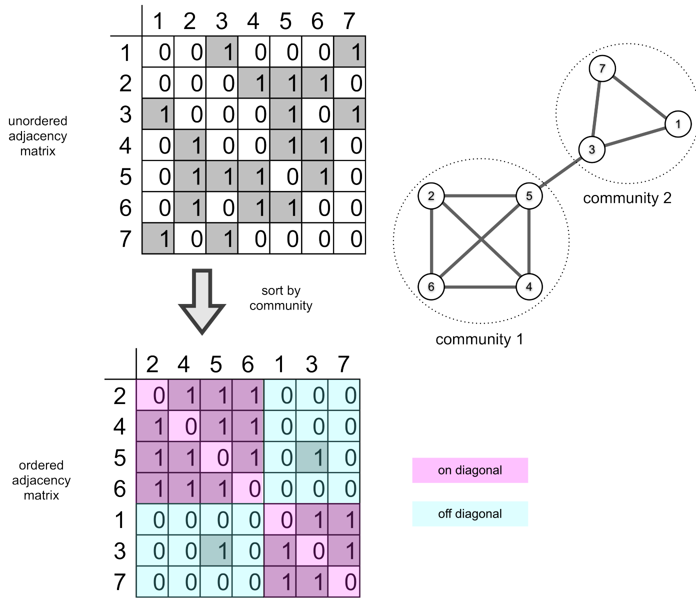

An adjacency matrix is a standard way of representing a network or graph where rows and columns are labeled nodes and the values of the matrix represent the strength of connection between nodes (weights). We use only undirected networks in this work, and therefore, all the adjacency matrices are symmetric. Consider the simple example to the left where all edges have the same weight and two communities exist.

Adjacency matrices can be ordered by community such that nodes within the same community are grouped together and on diagonal “blocks” are a visual representation of the communities; the off diagonal blocks should then contain less weight overall than the on diagonal blocks.

Communities

A community is a subgroup of nodes in a network that are more densely interconnected to each other than they are to the rest of the network. Two “community detection” procedures are used in MICC: 1) WGCNA is a state-of-the-art algorithm for detecting communities specifically in gene coexpression networks; the communities in this case are called modules. 2) Community detection methods based on the concept of a property called modularity are used on the module overlap graph as well as the functional genomic networks. For a detailed and comprehensive review of community detection methods and modularity, see Newman, 2012.

Top-level communities We use the term top-level communities to refer to communities in the module overlap graph identified with fast-greedy modularity maximization because this procedure yielded large, diffuse communities. (Fast-greedy modularity maximization has a strong bias for the size of communities it selects and is thought to find ‘low-resolution’ clusters in some cases.)

Bottom-level communities We use the term bottom-level communities to refer to communities in the module overlap graph identified using spinglass community detection. With our parameter choice, spinglass community detection is mathematically the same as the modularity maximization, but can find denser communities.

Subcommunities and consensus ordering. To reveal subcommunities (there is hierarchical community structure in the module overlap graph), we first sorted by top-level community label (modularity maximization), and then within each community we sorted by bottom-level label (spinglass/simulated annealing).

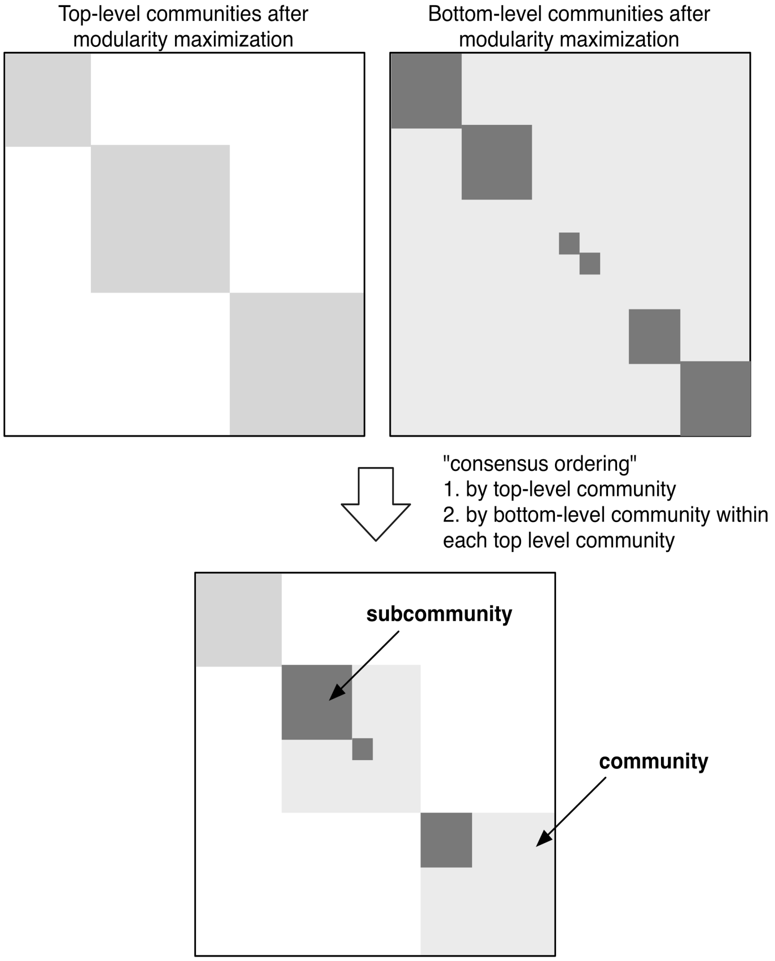

Principal components/Module eigengenes

Principal component analysis (PCA) is a procedure for simplifying high-dimensional data and summarizing it with fewer dimensions (e.g. to plot in two dimensions). In this paper, we used PCA to extract the first principal components of gene expression modules. The output of WGCNA is a set of gene clusters (modules). The gene expression of any gene within a module is very similar to (highly correlated with) that of any other gene in the same module. Thus, we can capture the major signal in the gene expression of the whole module by simply considering the *first* principal component of the module's full gene expression profile. This principal component is called the "module eigengene". The term eigengene is related to the fact that PCA is performed using eigenvalues/eigenvectors from linear algebra.

Hub

A hub is a node in a network that is extremely densely connected to the rest of the network (or a part of the network). Biological networks have a well-studied property (called “scale-free”) where most nodes (genes, proteins, etc.) are weakly connected to the rest of the network, but a small fraction of the nodes are extremely highly connected. These highly connected nodes are called hubs. It has been shown that hubs in biological networks are key molecules involved in their various biological functions, without which the system is severely impaired.

There are two types of hubs we consider in this paper. First, hubs in gene-gene coexpression networks are genes whose expression is highly correlated to the expression of many other genes. The module eigengenes from WGCNA represent theoretical genes that are the hubs of their respective module. The module eigengene, if it were a real gene, would be the most connected gene in the module. Genes that are very similar to an eigengene are therefore very highly correlated to the other genes within their module.

The other type of hub we consider are those in a functional genomic network. Again, in the GIANT networks nodes are genes, but the edges are (predicted) interactions between the genes. In this case, then, a hub is a gene that has a very high number of interactions with other genes.

Bridge

In this work, we refer to a gene (node) as a bridge if it is connected to (predicted to interact with) genes in multiple functional modules. We infer that these genes may function in more than one biological process represented in the network.

Centrality

Centrality is a measure of the relative importance of a node in a network; it can be and has been defined many different ways. For example, degree centrality, or simply degree, refers to the number nodes a node is linked to in a network. A node with relatively high degree may be said to be a hub.

Neighbors/neighborhood

A node’s first-degree neighbors in a network are those nodes to which it is directly connected by an edge. The term *neighborhood* of a node *n* refers to the set of nodes connected to (or adjacent to) *n*.

Mahoney JM, Taroni J, Martyanov V, Wood TA, Greene CS, Pioli PA, Hinchcliff ME, Whitfield ML (2015) Systems Level Analysis of Systemic Sclerosis Shows a Network of Immune and Profibrotic Pathways Connected with Genetic Polymorphisms. *PLoS Comput Biol* **11**: e1004005.

Newman ME (2012) Communities, modules and large-scale structure in networks. *Nature Physics* **8**: 25-31.
