# Supplementary material for: A novel multi-network approach reveals tissue-specific cellular modulators of fibrosis in systemic sclerosis
Source: Genome Med. 2017 Mar 23;9:27. doi: 10.1186/s13073-017-0417-1 (PMC5363043; doi:10.1186/s13073-017-0417-1)
Supplement: Supplementary file 30 — Supplementary figures and legends. (PDF 3174 kb) [file 13073_2017_417_MOESM30_ESM.pdf]

SUPPLEMENTAL FIGURE LEGENDS

Fig S1. Network view of consensus clusters 4A and 4B in the module overlap graph.

Fig S2. Density plot of correlation to respective module eigengene (kME). Tissue consensus genes have significantly higher kME and are therefore more 'hub-like' than non-consensus genes. Mann Whitney U, $p < 2.2 \times 10^{-16}$

Fig S3. Fully labeled version of the lung network (Fig 3A). This file is intended to be viewed digitally.

Fig S4. Fully labeled version of the differential adjacency matrix in Fig 4B. This file is intended to be viewed digitally.

Fig S5. The differential lung network. The highly lung-specific network (minus global network and skin network) contains functional modules.

Fig S6. The lung phagolysosome/ECM disassembly functional module is more highly connected in a macrophage-specific functional genomic network than expected. Global edge weights were subtracted from the lung and macrophage networks. The black and white matrix illustrates how much weight is within a community ("on-diagonal") or between communities ("off-diagonal"; how connected communities are to one another) by summing all edge weights in that block. We permuted the order of the adjacency matrix (edges) and assessed if the true weight within a community was more than random (red), less than random (blue), or no different than random (white). Note that there is more weight within communities in the (global subtracted) lung network than expected than random, which illustrates how the community detection algorithm utilized takes into account the expected distribution of edges/edge weights at random.

Fig S7. Analysis in Figure 5B repeated in Assassi, et al. skin dataset. Summarized expression values (mean standardized expression value) of gene sets (coexpression modules) upregulated in various MØ states. Overall, the differences between SSc skin and normal skin in this independent dataset agree with the findings in the Hinchcliff dataset, with the exception of the CL1 module; it is significantly upregulated in SSc skin in the Assassi dataset but not the Hinchcliff dataset.

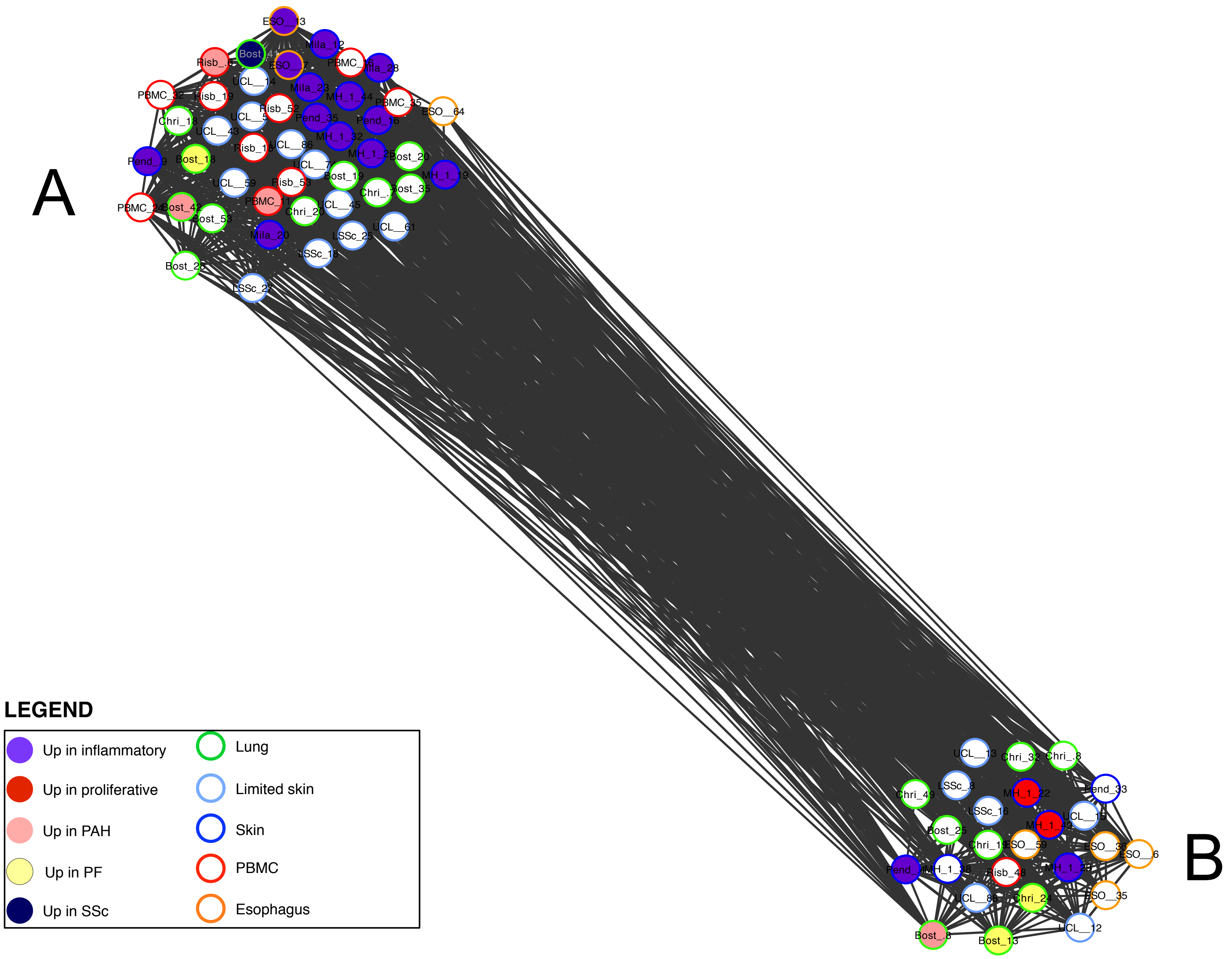
A
B
LEGEND
Up in inflammatory
Up in proliferative
Up in PAH
Up in PF
Up in SSc
Lung
Limited skin
Skin
PBMC
Esophagus

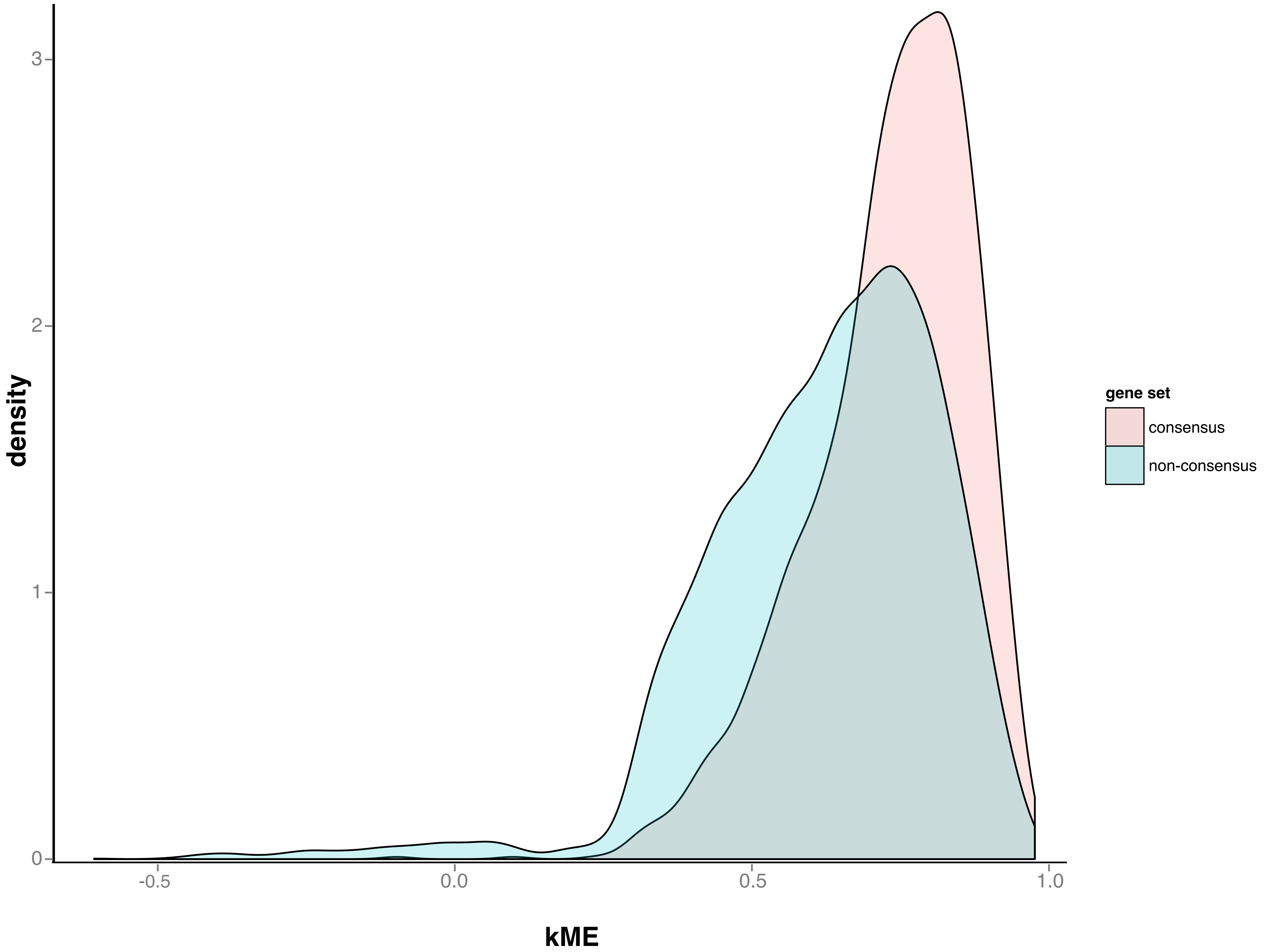

density
3
2
1
0
-0.5
0.0
0.5
1.0
kME
gene set
consensus
non-consensus

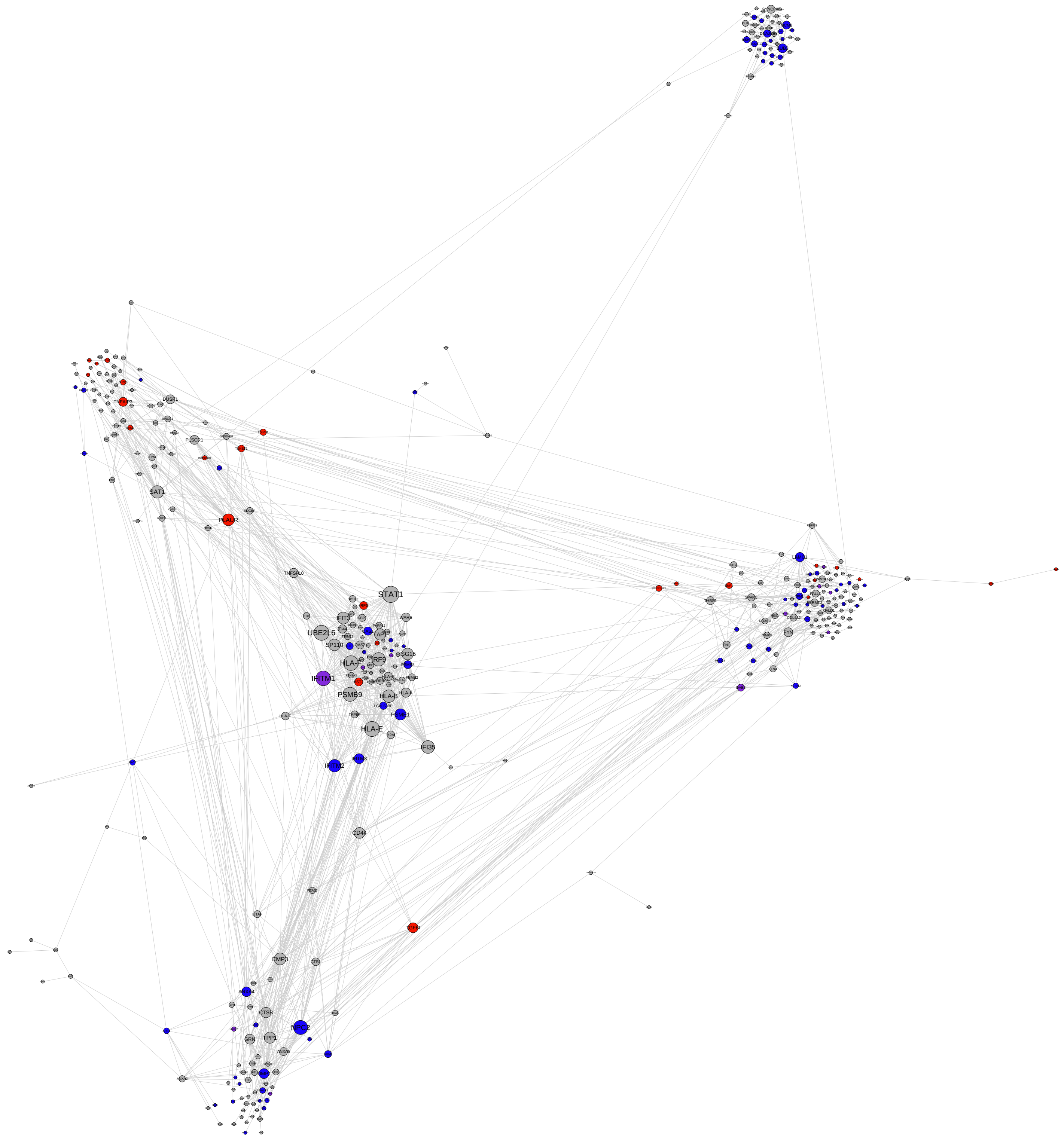
STAT1
UBE2L6
IFIT3
SP110
IRF9
ISG15
IFITM1
PSMB9
HLA-B
HLA-E
IFI35
CD44
EMP3
TPP1
GRN
SAT1
PLAUR
DUSP1
PLSCR1
TNFSF10
FYN

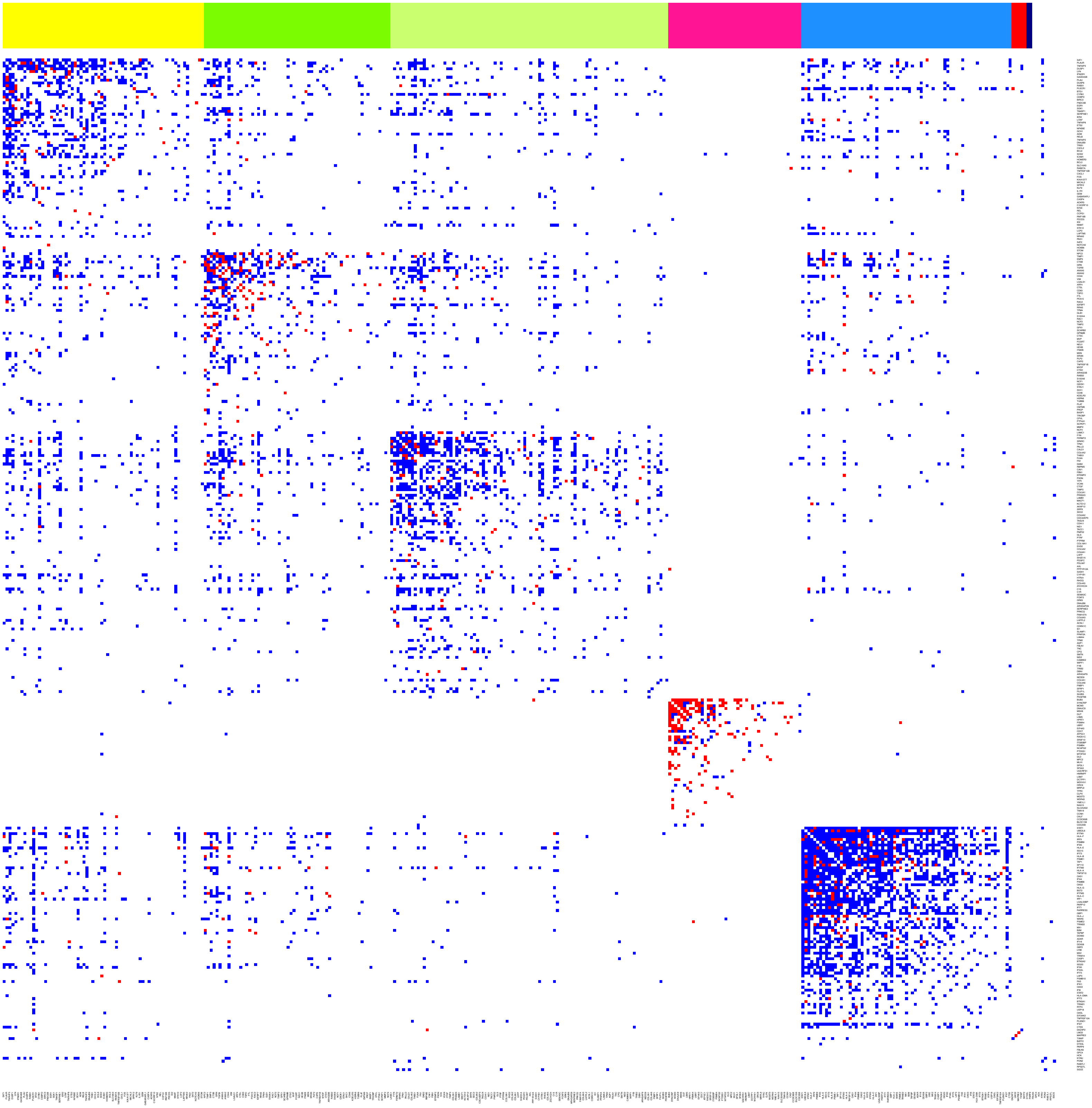

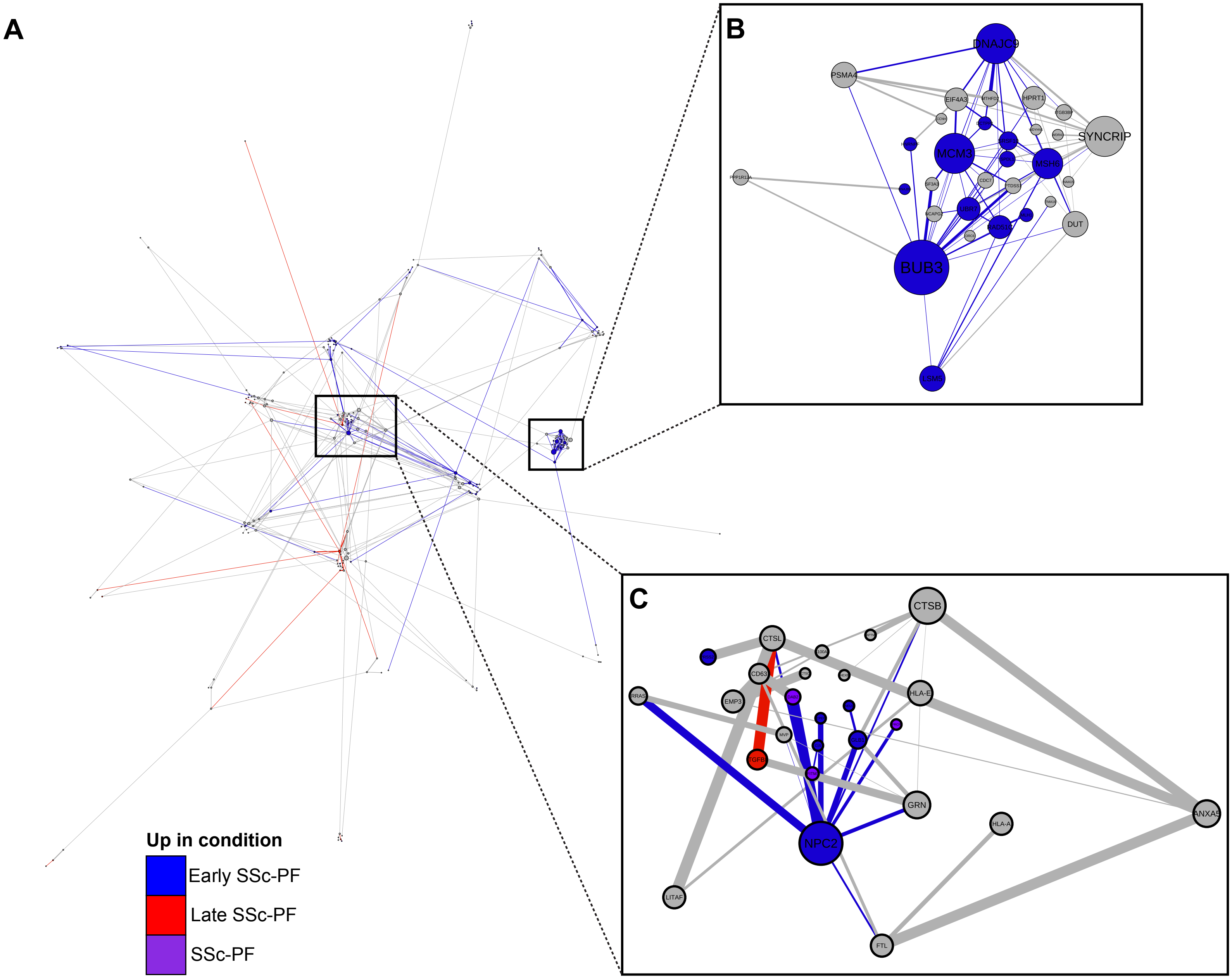

A
B
C
DNAJC9
PSMA4
EIF4A3
HPRT1
SYNCRIP
MCM3
MSH6
PPP1R12A
DUT
BUB3
LSM5
CTSB
CTSL
CD63
HLA-E
EMP3
RRAS
MVP
TGFB
GRN
ANXA5
HLA-A
NPC2
LITAF
FTL
Up in condition
Early SSc-PF
Late SSc-PF
SSc-PF

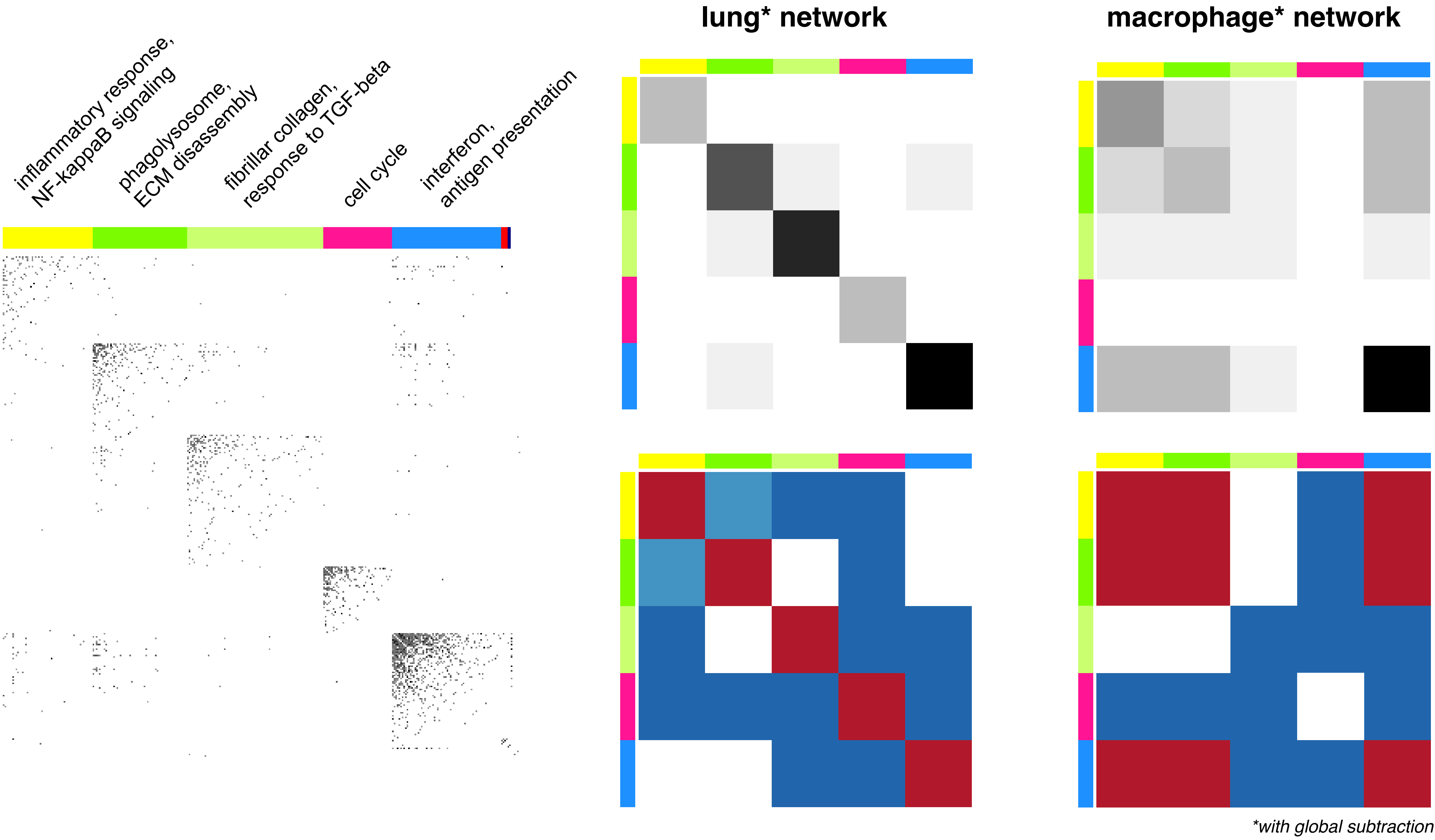
inflammatory response,
NF-kappaB signaling
phagolysosome,
ECM disassembly
fibrillar collagen,
response to TGF-beta
cell cycle
interferon,
antigen presentation
lung* network
macrophage* network
*with global subtraction

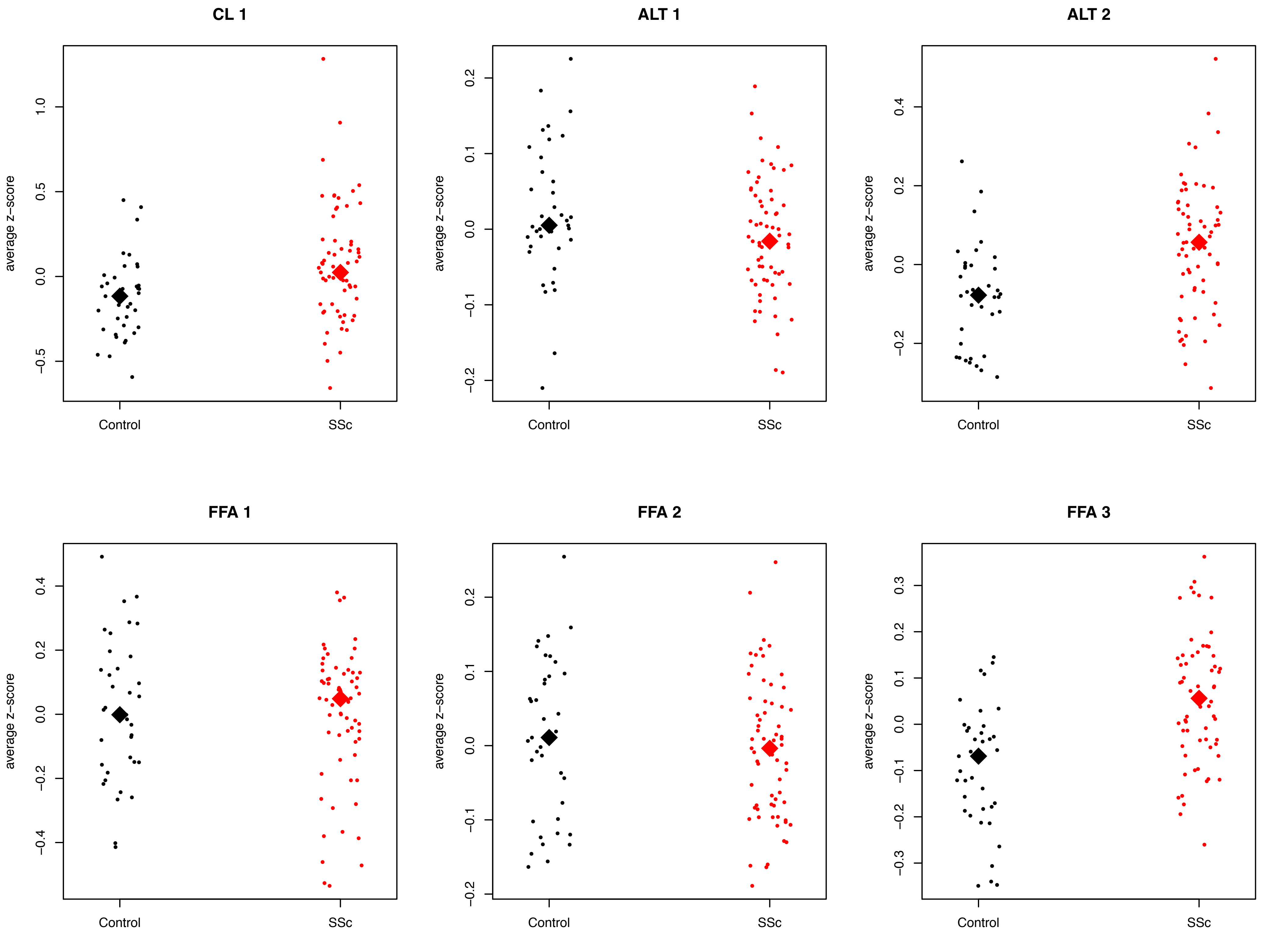
CL 1
ALT 1
ALT 2
FFA 1
FFA 2
FFA 3
average z−score
Control
SSc
1.0
0.5
0.0
−0.5
0.2
0.1
−0.1
−0.2
0.4
−0.4
0.3
−0.3
